# Production of pine sawdust biochar supporting phosphate-solubilizing bacteria as an alternative bioinoculant in *Allium cepa* L., culture

**DOI:** 10.1038/s41598-022-17106-1

**Published:** 2022-07-27

**Authors:** Andrea Blanco-Vargas, María A. Chacón-Buitrago, María C. Quintero-Duque, Raúl A. Poutou-Piñales, Lucía A. Díaz-Ariza, Carlos A. Devia-Castillo, Laura C. Castillo-Carvajal, Daniel Toledo-Aranda, Christiano da Conceição de Matos, Wilmar Olaya-González, Oswaldo Ramos-Monroy, Aura M. Pedroza-Rodríguez

**Affiliations:** 1 Laboratorio de Microbiología Ambiental y de Suelos, Unidad de Investigaciones Agropecuarias (UNIDIA), Departamento de Microbiología. Facultad de Ciencias, Pontificia Universidad Javeriana, Carrera 7ma No 43-82, Edifício 50 Lab. 106, 110-23 Bogotá D.C., Colombia; 2 Laboratorio de Biotecnología Molecular, Grupo de Biotecnología Ambiental e Industrial (GBAI), Departamento de Microbiología. Facultad de Ciencias. Pontificia, Universidad Javeriana, Bogotá, D.C., Colombia; 3 Laboratorio Asociaciones Suelo, Planta, Microorganismo (LAMIC). Grupo de Investigación en Agricultura Biológica, Departamento de Biología. Facultad de Ciencias. Pontificia, Universidad Javeriana, Bogotá, D.C., Colombia; 4 Facultad de Estudios Ambientales y Rurales, Departamento de Ecología y Territorio. Pontificia, Universidad Javeriana, Bogotá, D.C., Colombia; 5 Facultad de Ciencias de la Salud, Universidad Anáhuac Campus Norte, México, D.F., Mexico; 6 Departamento de Ciências Agrárias e Naturais, Universidade do Estado de Minas Gerais, Ituiutaba, Minas Gerais Brazil; 7 Instituto Politécnico Nacional, Escuela Nacional de Ciencias Biológicas, Prolongación de Carpio y Plan de Ayala S/N, Col. Santo Tomás, 11340 México CDMX, Mexico

**Keywords:** Biotechnology, Microbiology, Materials science

## Abstract

We produced and characterised biochar made from Caribbean pine sawdust as raw material. The biochar (BC_500_) was used as biocompatible support to co-inoculate phosphate solubilizing bacteria (PSB) (BC_500_/PSB) on *Allium cepa* L., plants at a greenhouse scale for four months. The three biomaterials study included proximate analysis, elemental analysis, aromaticity analysis, scanning electron microscopy, Fourier transform infrared spectroscopy (FTIR), adsorption studies at different pH and PSB stability as a function of time. The results indicated that BC_500_ is suitable as organic support or solid matrix to maintain the viability of PSB able to solubilise P from phosphate rock (PR). The biofertilizer (BC_500_/PSB) allows increasing germination, seedling growth, nutrient assimilation, and growth of *Allium cepa* L., because PSB immobilised on BC_500_ promoted nutrient mobilisation, particularly P, during cultivation of *Allium cepa* L., at pots scale. The two treatments to evaluate the biofertilizer (BC_500_/PSB) showed the highest concentrations of total P with 1.25 ± 0.13 and 1.38 ± 0.14 mg bulb^−1^ in *A. cepa* L. This work presents the benefits of a new product based on bacteria naturally associated with onion and an organic material (BC_500_) serving as a bacterial carrier that increases the adsorption area of highly reactive nutrients, reducing their leaching or precipitation with other nutrients and fixation to the solid matrix of the soil.

## Introduction

Colombia is a forestry vocation country with the potential for implementing commercial reforestation programmes. The geostrategic location of Colombia is favourable for the trade of agroforestry products^1^. For commercial purposes, the most cultivated genera and species are *Pinus caribaea*, *Tabebuia rosea*, *Tectona grandis* and *Eucalyptus pellita*^2–4^. For their production, forestry companies implement the entire production process, including high-quality seeds or clones, propagation of plant material in nurseries, forest plantations and harvesting areas^3–5^. At the harvesting stage, large quantities of solid waste rich in lignocellulosic biomass (sawdust, shavings, bark, leaves or stems) are generated and could be up to 50% of the processed wood^6–8^. The degradation of this waste is slow as it consists of lignin, cellulose, and hemicellulose^9,10^. These polymers are complex, resistant, hydrophobic, and their biological transformation (landfill and composting processes) is slow^7,11–13^; resulting in a high percentage of these wastes not being used appropriately or being used raw (unprocessed) in agro-industrial processes as filler mixes for compost production^11,14^, insulating material for poultry, pig and livestock rearing farms^15–17^ and are used as planting substrates for the propagation of plant material in forest nurseries^6,7,18^.

Although the use of these raw or partially transformed agro-industrial by-products is widespread worldwide, other alternatives can be evaluated, such as thermal conversion or pyrolysis under reduced conditions or in the absence of oxygen. Through this physical process, new products such as biochar, oils, gases, and volatile compounds, among others, can be obtained^19–22^. Pine sawdust is one of the most used materials due to its cheapness, is found in large quantities and could be used for different purposes^19,23,24^.

In general, biochar offers a high surface area, porosity, nutrients associated with the initial biomass and capacity to retain water and microorganisms^25–30^, successfully used in agriculture as an organic amendment or organic soil conditioner, because it improves soil structural stability, porosity, hydraulic conductivity, soil aeration and cation exchange capacity^26,31,32^; generating an increase in nutrient availability, soil fertility and therefore a beneficial effect on different crops^31^. Additionally, due to its high porosity, biochar offers a favourable niche for soil microorganisms or added as biological inoculants called plant growth-promoting rhizobacteria (PGPR), allowing them to remain viable and metabolically active for a long time^25,33–36^. PGPR have direct and indirect mechanisms by which they promote plant growth. Direct mechanism includes biofertiliser activity, root growth stimulation, rhizobia-remediation, and stress control in plants^25,32,36–38^. Indirect mechanisms include biological control such as antibiosis, competition, and induction of systemic resistance in plants^25,39–41^.

When using biochar as biocompatible organic support for the formulation for co-inoculation of beneficial microorganisms, a dual-purpose biomaterial is added to the soil, incorporating a stable and slow-release form of carbon, if compared to the incorporation of fresh plant residues^26,42^. On the other hand, biochar is support, protecting microorganisms from environmental conditions effect, such as temperature, ultraviolet radiation, desiccation, predation by soil microorganisms^25,26,34^. Among the microorganisms that have been combined with biochar and added to soils are nitrogen-fixing bacteria, plant growth-promoting bacteria, biocontrol microorganisms, and phosphate solubilising bacteria (PSB)^25,33,34,37^.

PSB co-inoculated in biochar can solubilise unavailable forms of inorganic phosphorus or mineralise unavailable organic phosphorus, part of organic matter^43^. For solubilisation, PSB uses different mechanisms such as the production of organic acids, siderophores, and the release of protons, among others^37,44^. In connection with mineralisation, acid phosphatase (EC 3.1.3.2) and alkaline phosphatase (EC 3.1.3.1) enzymes can be produced^25,43^. Due to these two mechanisms, available inorganic forms of phosphorus are released into the soil as orthophosphate ions (H_2_PO_4_^−^, HPO_4_^2^ and PO_4_^3^) for plants and soil microorganisms^37,44,45^.

In the production of vegetables such as *Allium cepa* L. (bulb onion), phosphorus limitation in the different stages of production (seedbed and field) is a nutritional factor affecting crop growth, productivity, and yield^33,37,45^. To supply this nutritional requirement, traditional and intensive agricultural practices apply large doses of chemical fertilisers based on nitrogen, phosphorus, and potassium (N/P/K), which in the long term affect the quality and quantity of soil organic matter and degrade soil fertility^25,43,44,46–48^.

For these reasons, researchers seek sustainable biotechnological alternatives such as the use of biochar/PSB^25,33,36,37^. This biomaterial could be used in a mixture with inorganic (N/P/K) or organic fertilisers (compost and vermicompost) in different doses and thus decrease the excessive use of chemical fertilisers as sole sources of phosphorus ^31,33,42,45^. It becomes an environmentally friendly strategy, as it integrates the use, conversion and exploitation of a forest residue with the application of beneficial microorganisms (PSB) in vegetables such as *Allium cepa* L.^25,34,37,44,45,49^.

In this article, BC_500_ serve as organic support to co-inoculate a consortium of PSB, composed of *Pseudomonas* sp., *Serratia* sp., and *Kosakonia* sp. Furthermore, the effect of BC_500_/PSB on *Allium cepa* L., growth was studied for four months at the greenhouse scale.

## Materials and methods

### Biochar production

Caribbean pine sawdust (CPS) from a Colombian wood processing company was used for biochar production. The CPS was sieved sequentially (using sieves No 10, 12 and 20) to obtain a particle size of about 4–5 ± 1 mm. It was dried for 24 h at 70 ± 2 °C in a HACEB electric oven and preserved in a hermetically sealed plastic bag to control humidity^33^.

A 250 g aluminium-container with 100 ± 5 g of dried CPS was placed inside a 2.5 L 3 M® brand anaerobic hood, containing a 3 M Anaerogen sachet. This system allows to displaced O_2_ after incubation for 12 h at 19 ± 2 °C. Subsequently, each vessel was transferred to a 20 L Labtech™ flask and heat-treated at 500 ± 5 °C for 1 h by using a heating rate of 10 °C min^−1^. Later the BC_500_ was removed from the flask and placed back in anaerobic hoods to prevent it from acquiring moisture^50^.

### Characterisation of BC_500_

The pH and a moisture percentage of all, the Caribbean pine sawdust (CPS), the biochar alone (BC_500_) and the biochar co-inoculated with PSB (BC_500_/PSB) were determined following the methodology reported by Colombian Technical Standard 5167^51,52^. As part of the proximate analysis, the following were determined: percentage volatile carbon (VC)^53,54^, ash percentage (Ash)^53,54^, fixed carbon percentage (FC)^53,54^, total organic carbon percentage by ignition (TOC)^33^, and biochar yield^30^.

The analysis of the elements (C, O, H, N and S) was done by the complete and instantaneous oxidation of the sample by using combustion with pure oxygen at 1000 ± 10 °C. The equipment used was a Thermo Flash® 2000, with a reactor temperature of 950 °C with He and O_2_ flow rates of 140 and 250 mL min^-1^, respectively. Complementary analyses allow determining the C/O, H/O and (O + N)/C ratios.

The surface characteristics and morphology of CPS, BC_500_ and BC_500_/PSB were observed by scanning electron microscopy coupled to energy dispersive X-ray (SEM/EDS), with a JEOL scanning electron microscope, model JSM 6490-LV with power between 10–20 kV and magnifications between 150 × and 17,000 × ^33,45^.

To identify the chemical functional groups of CPS, BC_500_ and BC_500_/PSB, a Fourier array infrared spectroscopy analysis was carried out by using a Shimadzu IR Prestige-21 spectrophotometer with an ATR module; the parameters set up were Measurement Mode: % transmittance, Apodization: Happ-Genzel, No. of scans: 40, Resolution: 4.0, Range (cm^−1^): 600–4000^33^.

### Adsorption studies of PSB and orthophosphates to BC_500_

The pH effect on the adsorption of PSB to BC_500_ was done by following the reported methodology^55^; the initial PSB concentration was 1.0 × 10^7^ ± 1.0 × 10^1^ CFU mL^−1^. In 250 mL flasks containing 100 mL of PSB at different pH (3.0, 5.0 and 7.0) adjusted with 0.5 M sodium hydroxide or 0.5 M hydrochloric acid, were added with 2.0 g of biochar, and shaken at 120 rpm for 60 min at 19 ± 2 °C in a Heidolp brand horizontal shaker. Samples taken were at baseline and every 10 min for decimal dilutions and 0.1 mL of each dilution was seeded on Petri dishes containing brain heart infusion (BHI) agar^45^. Using the count data expressed as logarithm (Log_10_) of CFU g^−1^ adsorbed to BC_500_, the value of *qe* (number of cells adsorbed per g of BC_500_) was calculated using Eq. 1.

1 $$qe = \frac{{V\left( {C_{0} - C_{f} } \right)}}{X}$$

where: *V* = volume of solution (L), *C*_*f*_ = final cell concentration (Log_10_ CFU g^−1^), *C*_*o*_ = initial cell concentration in solution (Log_10_ CFU g^−1^), *X* = g of BC_500_ in dry weight.

To determine the pH effect (3.0, 5.0 and 7.0) on orthophosphate adsorption, we use 100 mL flasks containing 50 mL of a standard PO_4_-P solution (100 mg L^−1^) and 1.0 g of BC_500_ (in triplicate). The flasks were shaken at 150 rpm and 25 ± 2 °C for 120 h in a shaker (Scientific CVP-500). Samples taken were at baseline, at 20, 40 and 80 min, also at 2, 24, 48 and 120 h. The molybdate-vanadate colourimetric technique^56^ allows for determining the PO_4_-P concentration (mg L^−1^). These concentrations serve to calculate the *qe* value (amount of PO_4_-P absorbed/g BC_500_) using Eq. 1, but with modified response variables concerning PO_4_-P. With the results of *qe* obtained from PSB and orthophosphates, were calculated the adsorption constants by applying the pseudo-second-order linearisation models^57^ and Elovich^58^.

### Co-inoculation of phosphate-solubilising bacteria to BC_500_

PSB (*Serratia* sp., *Pseudomonas* sp., and *Kosakonia* sp.) previously isolated in our research group^45^ were cultured following the methodology reported^55^. Quality control involves counting on MT11B agar, measurement of pH and orthophosphate concentration by using Merck's Spectrocuant Phosphate Test Kit (MQuant Test Phosphates)^59^. Concentrated PSB were dosed to obtain a concentration of 1.0 × 10^7^ ± 1.0 × 10^1^ CFU mL^−1^ at pH 3.5^51,52^. 200 g of BC_500_ were manually mixed with 19 mL of the PSB (1.0 × 10^7^ CFU mL^-1^) until obtained a hydrated material. Following, the vessels containing BC_500_/PSB were incubated for 24 h at 30 ± 2 °C in a Memmert brand incubator, following the methodology of patent # WO 2014/167,409 Al^18^.

### Effect of BC_500_/PSB on the growth of *Allium cepa* L.

The soil used in this experiment came from the San Juan farm located in the village of “Punta Larga” in the municipality of “Nobsa” in the Department of “Boyacá, Colombia” (5º47′03,5 "N 72°58′52,6 "W). The soil was dried at 40 ºC for 48 h in a HACEB electric oven. Later, it was disaggregated with a rubber mallet and sieved with a No. 20 sieve to get particles about 4–5 ± 1 mm, and sterilised three times in an autoclave for 15 min, 1.2 atm., and 121 °C, leaving intervals of 24 h between each cycle.

The study was performed in 1 kg plastic pots; containing 850 g of agricultural soil alone or combined with two concentrations of BC_500_/PSB (2.0 or 5.0% w/w). Previously transplantation a single dose of Abundagro fertilisation was applied. The amount of Abundagro added was the equivalent of adding 35 kg ha^−1^, the composition of this organic mineral fertilizer is 2.8 g Kg^−1^ organic nitrogen, 80 g Kg^−1^ assimilable phosphorus, 23 g Kg^−1^ potassium, 240 g Kg^−1^ calcium, 11. 7 g Kg^−1^ magnesium, 0.1 g Kg^−1^ boron, 0.02 g Kg^−1^ zinc, 60 g Kg^−1^ iron, 0.2 g Kg^−1^ silicon, 0.02 g Kg^−1^ copper, 80 g Kg^−1^ organic matter, 26 g Kg^−1^ additional nitrogen source and pH of 7.35 ± 0.2.

*Allium cepa* L*.,* is not a plant native to Colombia; it is a plant species introduced many times before for commercial production. Therefore, no collection permit or licence was required to purchase them. Seeds of *Allium cepa* L., were purchased from local farm shops. Colombia is a large producer of this vegetable.

Plants obtained from standard Granex variety seeds sown in peat (Impulsemillas) were transplanted 20 days after sowing in seedbeds. A randomised complete block experimental design was used, with four blocks, six treatments, and 40 seedlings per treatment, for a total of 240 experimental units (Table 1). Irrigation with potable water was carried out every other day at field capacity (100 mL). Before starting the assays, it was done the physical, chemical and microbiological characterisation, for BC_500_, BC_500_/PSB, soil with BC_500_/PSB at 5.0% (w/w) (T1), soil with BC_500_/PSB at 2.0% (w/w) (T2), soil with BC_500_ at 2.0% (T4), and soil with BC_500_ at 5.0% (w/w) (T3) (Supplementary Material S1)^60^.

**Table 1:** Treatments evaluated during the pot experiment.

Treatment	Abundagro® dosification, biofertilizer (BC_500_/PSB) and biochar (BC_500_)
T1	Soil + Abundagro 70 kg ha^−1^ + biofertilizer (BC_500_/PSB) at 5.0% (w/w)
T2	Soil + Abundagro 70 kg ha^−1^ + biofertilizer (BC_500_/PSB) at 2.0% (w/w)
T3	Soil + Abundagro 70 kg ha^−1^ + biochar (BC_500_) at 5.0% (w/w)
T4	Soil + Abundagro 70 kg ha^−1^ + biochar (BC_500_) at 2.0% (w/w)
T5	Soil + Abundagro 70 kg ha^-1^
T6	100% Soil

Four months after transplanting of plants, the bulb fresh weight (BFW) (mg), the bulb diameter (BD) (mm), the bulb height (BH), (mm), the root fresh weight (RFW) (mg), the root length (RL) (mm), the leaf fresh weight (LFW) (mg), and the total fresh weight (TFW) were measured^61–63^. Subsequently, plant material was dried at 40 ± 2 °C for 15 days^64^, to determine bulb- (BDW) (mg), root- (RDW), leaf- (LDW) and total-dry weight (TDW)^61–63^.

An external reference laboratory (AGRILAB) performed the complete nutritional analysis of the plant material and soils; for this purpose, were randomly selected 2 bulbs of *A. cepa* L., from each treatment with their respective soil (8 bulbs and 8 soil samples per treatment). The final soil concentration of nutrients was expressed as the variation of the soil characteristics; calculated by subtracting the value obtained at the end of the biofertilizer evaluation from the initial value for each of the samples evaluated. Negative values of some variables indicated an increase in the content of the nutrient or variable in the soil at the end of the experiment (Table 5), concerning the initial content before transplanting (Supplementary Material S1). Statistical analysis of all the variables involves the ANOVA and Tukey's multiple comparisons test, using the statistical programmes R (StatR, R Wizard platform version 2.0) and Minitab (Minitab 18. Ink. 2018. version 18.0).

## Results

### Characterisation of CPS and BC_500_ sawdust

Table 2 shows the results of the CPS; the moisture content was 7.0 ± 0.31%, with a bulk density of 0.22 ± 0.09 g cm^-3^, the porosity of 57.5 ± 5.8% and a sieved particle size of 5.0 ± 1 mm. Concerning the chemical variables, the pH was 3.7 ± 0.08, and the electrical conductivity was 0.53 ± 0.02 dS cm^-1^. Other results related to the percentage of lignin (40.9%), cellulose (39%), hemicellulose (15%), elements, the sum of cations and the sum of anions in the SCP appear in the Supplementary Material S2.

**Table 2:** Proximate and elemental analysis for Caribbean pine sawdust (CPS), biochar produced at 500 °C 1/h (BC_500_) and co-inoculation process with PSB (BC_500_/PSB).

Parameters	Caribean Pine Sawdust (CPS)	Biochar (BC_500_)	Biochar co-inoculated with PSB (BC_500_/PSB)
**Basic Properties**			
Moisture (%)	7.0 ± 0.31	3.6 ± 0.51	99 ± 2
Apparent Density (g cm^-3^)	0.22 ± 0.09	0.43 ± 0.51	0.42 ± 0.1
Porosity (%)	57.5 ± 5.8	67 ± 1	64.3 ± 1.1
Size particle (mm)	5.0 ± 1	3.0 ± 0.7	3.0 ± 1
pH	3.7 ± 0.8	7.1 ± 0.6	6.4 ± 0.5
Electrical Coductivity dS cm^-1^	0.53 ± 0.02	ND	ND
**Proximate analysis (wt.%, wet basis)**			
TOC (%)	57.89 ± 0.15	50.9 ± 2.2	54 ± 3
CF (%)	14.15 ± 0.21	26 ± 2	23 ± 2
CV (%)	80.68 ± 0.31	71.6 ± 2.4	75 ± 3
Ash (%)	5.17 ± 0.37	2.4 ± 0.9	2.0 ± 0.9
Yield _biochar_ (%)	NA	19 ± 0.7	NA
**Elemental analysis (wt.%, dry basis)**			
C (%)	48.16	71.04	25.12
O (%)	45.87	26.9	73.61
H (%)	5.89	1.74	1.23
N (%)	0.08	0.32	0.04
**Atomic ratio**			
H/C ratio	0.12	0.02	0.05
O/C ratio	0.95	0.381	2.93
(O + N)/C	0.954	0.383	2.931
**Bacteria co-inoculated in BC**_**500**_			
Total PSB count UFC g^-1^	NSD	NSD	4.0 × 10^5^ ± 1.0 × 10^2^
*Pseudomona* sp.	NSD	NSD	3.0 × 10^5^ ± 1.0 × 10^1^
*Kosakonia* sp.	NSD	NSD	1.3 × 10^5^ ± 1.0 × 10^1^
*Serratia* sp.	NSD	NSD	2.6 × 10^5^ ± 1.0 × 10^2^

* NSD: Not determined.

Concerning BC_500_, a smaller size material (3.0 ± 0.7 mm) than CPS was obtained, observing small, elongated, thin and black chips. The percentage of moisture decreased for the CPS, obtaining 3.6 ± 0.51%. An increase in density, porosity percentage and pH were also observed (0.43 ± 0.51 g cm^-3^, 67 ± 1% and 7.1 ± 0.6 respectively) (Table 2).

Proximate analysis shows TOC decreased in BC_500_ (50.9 ± 2.2%), when compared to SCP (57.89 ± 0.15%). The CF was 26 ± 2%, the VC 71.6 ± 2.4%, the ash content 2.4 ± 0.9% and the biochar yield was 19 ± 0.7% (Table 2). Classification results of BC_500_ indicated that it is a type II biochar (TOC > 30 and lower than 60%) ^65^.

Elemental analyses of CPS and BC_500_ are in Table 2. An increase in carbon contains about 71.05% was observed for BC_500_ compared to CPS; oxygen and hydrogen content decreased compared to the carbon content (26.9 and 1.74%). Regarding the atomic ratios of H/C, O/C and (O + N)/C, a decrease occurred for BC_500_ (0.02, 0.381 and 0.383 for H/O, O/C and (O + N)/C, respectively) compared to CPS (0.12, 0.95 and 0.954, respectively); indicating changes in aromaticity, stability, polarity, and functionality of the new material (Table 2).

### Infrared spectroscopy with Fourier arrays

**Figure 1:**
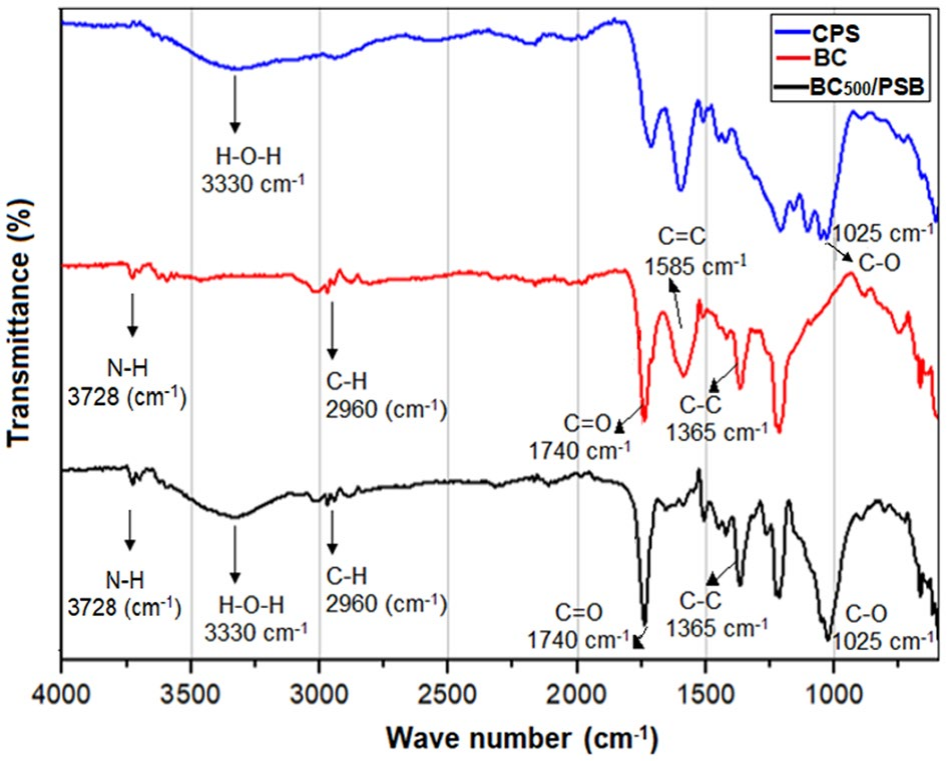
Fourier transformed infrared spectroscopy (FITR) for Caribe Pine sawdust (CPS), (Blue line), biochar (BC_500_), (Redline) and biochar co-inoculated with PSB (BC_500_/PSB), (Blackline).

The information obtained from the FTIR analysis indicated that CPS, BC_500_ and BC_500_/PSB presented organic characteristics, observing in each of the samples the presence of vibration bands associated with organic functional groups. In the CPS (Blue line), well-defined signals appeared at 1025 cm^-1^, 1585 cm^-1^ and 1740 cm^-1^, corresponding to symmetrical C–O single bonds, C=C double bonds and C=O double bonds. Additionally, at approximately 3330 cm^-1^ broadband, associated with O–H bonds was formed. In the BC (Redline), the signals of single C–O bonds (1025 cm^-1^) and O–H bonds (3330 cm^-1^) disappeared after heat treatment at 500 ºC for one h. Also, the heat treatment helped to broaden and define some signals that were in the CPS. The bands related to C=C bonds (1585 cm^-1^) and C=O double bonds (1740 cm^-1^) stand out. On the other hand, the thermal treatment favoured new bands formation that is not present in the CPS. Among those highlighted is the signal at 1365 cm^-1^ which corresponds to C–C bonds and the signal at 2960 cm^-1^ related to C-H bonds (Fig. 1).

After co-inoculation of PBS to biochar (BC_500_/PSB) (Blackline), appeared changes in the FTIR spectra. The signals at 1025 cm^-1^ (C–O bonds) and 3330 cm^-1^ (O–H bonds) do not appear in BC and appeared in BC_500_/PSB. Signals at 1365 cm^-1^ (C–C bonds), 1740 cm^-1^ (C=O bonds) and 2960 cm^-1^ (C–H bonds) were like BC_500_ without co-inoculation. Finally, in BC_500_/PSB, the signal at 1585 cm^-1^ disappears upon PSB co-inoculation. These results suggest that heat-treatment at 500 °C for 1 h generates more changes in the functional groups on the BC surface than co-inoculation with PSB (Fig. 1).

### Scanning electron microscopy for pine sawdust and biochar

**Figure 2:**
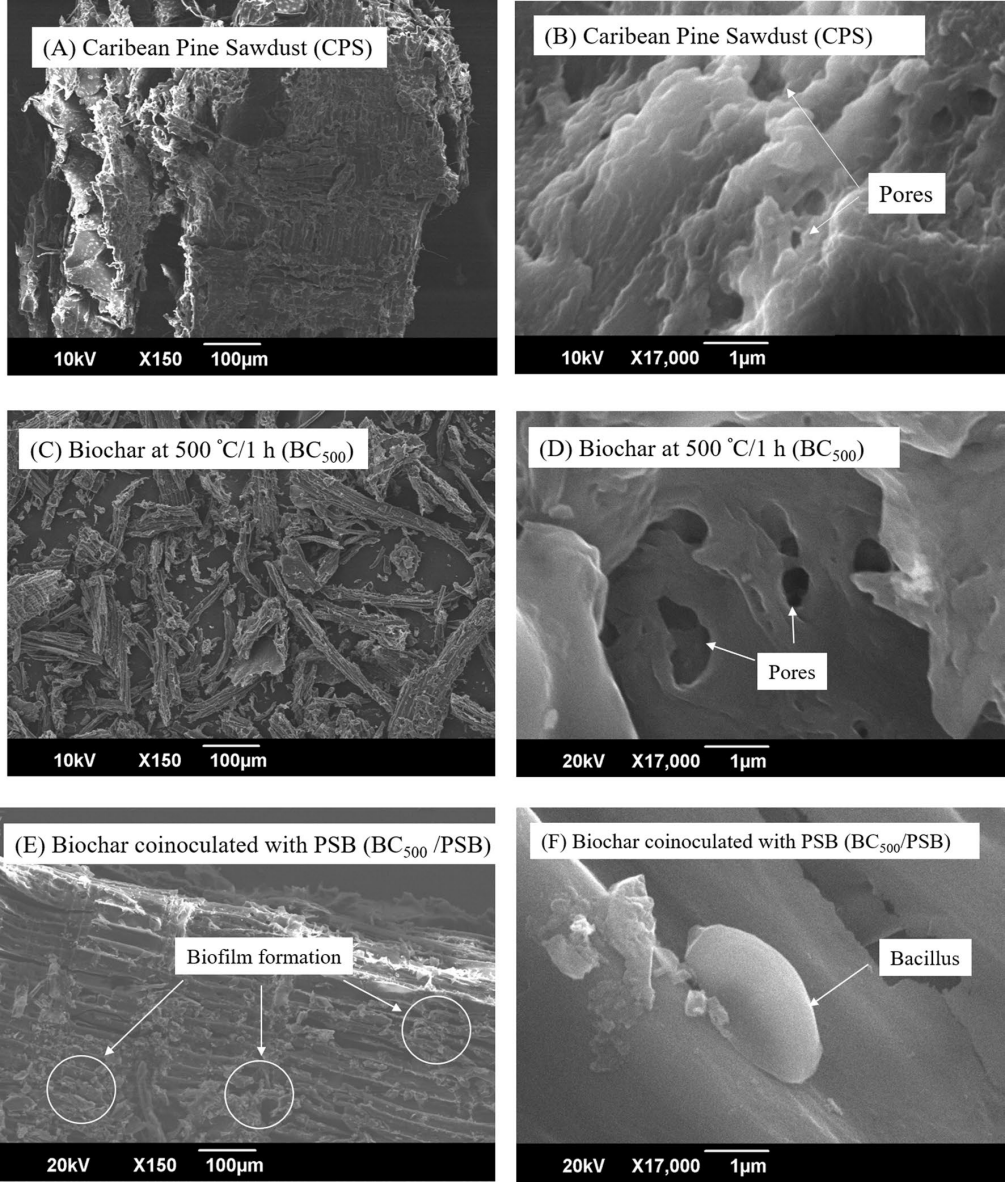
Morphological characteristics of the material. (**A**) Caribean Pine Sawdust (CPS) at SEM 150x. (**B**) Caribean Pine Sawdust (CPS) at SEM 17000x. (**C**) Biochar (BC_500_) at 500 °C/1 h, SEM 150x (**D**) Biochar (BC_500_) at 500 °C/1 h, SEM 17000x. (**E**) Biochar co-inoculated with PSB (BC_500_/PSB), SEM 150x. (**F**) Biochar co-inoculated with PSB (BC_500_/PSB), SEM 17000x.

Observation of the CPS at low magnification (150x) showed an irregular surface and shape, with cavities of different sizes, smaller than 100 µm. At higher magnification (17000x), a more homogeneous surface, layers and porous were observed (Figs. 2A, B). BC_500_ observation (150x) showed polymorphic and polydisperse fragments, with predominantly elongated and thin shapes, contrasting with the initial SCP observation (Fig. 2C). Upon heat treatment at 500 °C, the surface of BC_500_ became smoother than the CPS, with more pores of different sizes and more defined edges (Fig. 2D), which could result from displacement of water, volatile compounds, and oxygen at 500 °C (Fig. 2D). Figure 2E and F, correspond to the BC after co-inoculation of the PSB, at low magnification, the formation of a biofilm distributed on the surface of the biochar is observed, which suggests that the pH of the culture medium favoured the adsorption of the bacteria to the porous surface (Fig. 2E). Figure 2D shows the characteristic morphology of one of the short bacilli parts of the PSB. In addition, the integrity of the cell wall and the shape diffusion demonstrated that co-inoculation and secondary reactivation did not exert a negative effect on the bacilli.

### Adsorption studies for PSB

The pyrolysis temperature affects the chemical properties of BC_500_ compared to CPS, which had an acidic pH (3.7 ± 0.8). BC_500_ had a pH of 7.1 ± 0.6, and the zero-loading point (pHzpc) was 4.1 ± 0.8^43^. These results indicate that BC_500_ without microorganisms is negatively charged (pH 7.0 > pHzpc). Under these conditions, different interactions could occur between BC_500_ and adsorbates (bacteria and orthophosphates)^66^.

**Figure 3:**
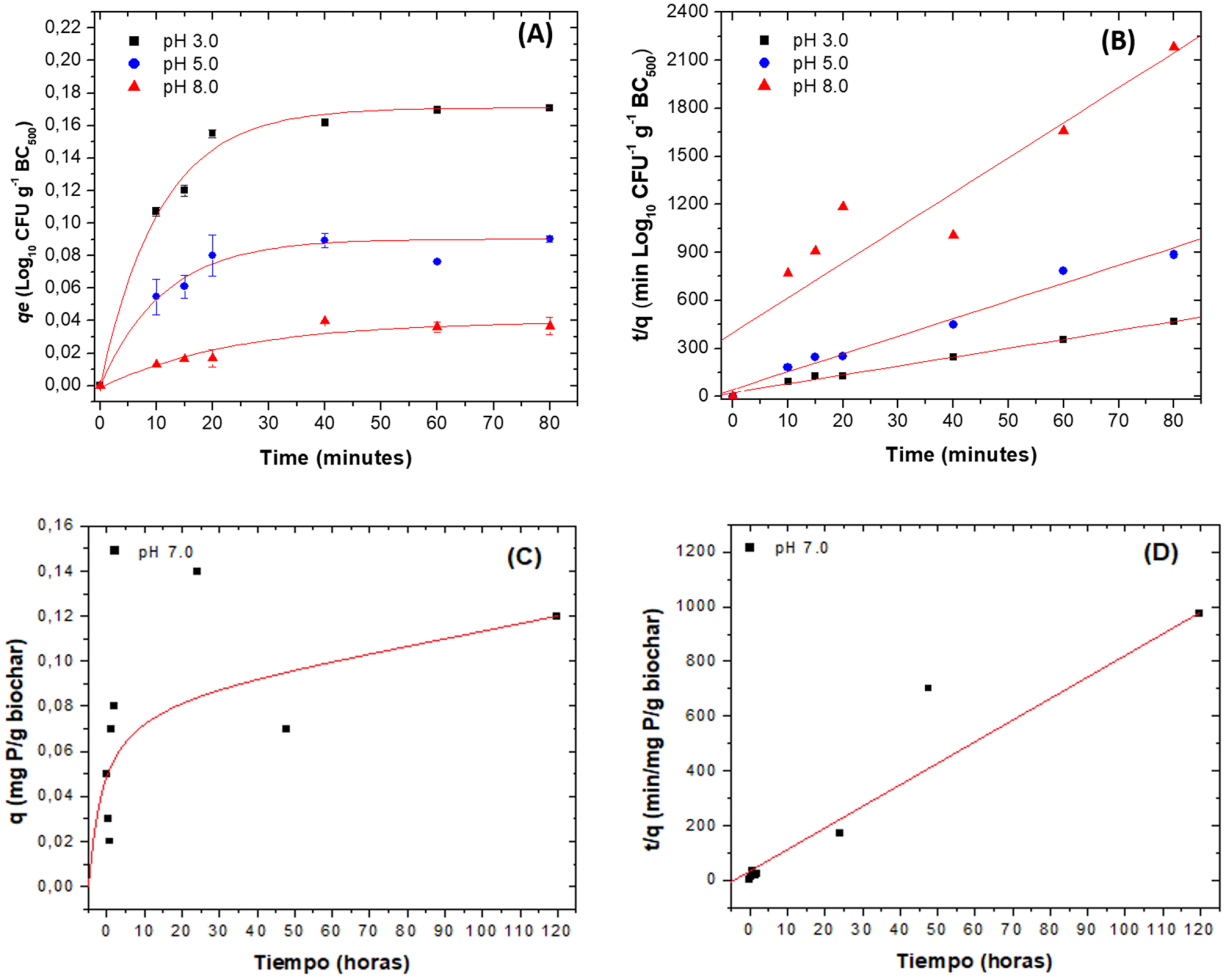
Adsorption studies. (**A**) *qe* value as a function of time at different pH for PSB. (**B**) Pseudo-second-order model for PSBs. (**C**) *qe* value as a function of time at pH 7.0 for orthophosphate ions. (**D**) Pseudo-second-order model for orthophosphate ions. Results are the average of three replicates with their respective deviation.

For the adsorption experiment with the PSBs, microorganisms adsorbed rapidly from the first minutes of contact and reached the adsorption/desorption equilibrium within 20 ± 10 min, for pH 3.0 and 5.0. At pH 8.0, a longer contact time takes to reach equilibrium (40–60 min). Concerning the effect of pH, significant differences were observed for pH; at pH values of 3.0, the highest adsorption of PSBs to BC_500_ was improved, obtaining a value of *qe* equal to 0.141 Log_10_ CFU g^-1^ BC_500_ (*p* < 0.0001). At pH 5.0 the *qe* value was 0.044 Log_10_ CFU g^-1^ BC and at pH 8.0 the lowest value was 0.019 CFU g^-1^ (Fig. 3A).

Pseudo-second-order model best described the adsorption process of the PSBs to BC_500_, obtaining R^2^ of 0.9991, 0.9792 and 0.8587, for pH 3.0, 5.0 and 8.0 (Fig. 3B). The calculated *qe* values for the three pH were like the experimental *qe* values, and the pseudo-second-order adsorption constants for pH 3.0 and 5.0 were higher (0.240 and 0.256 g Log_10_ CFU^-1^ min^-1^) than for pH 8.0 (0.055 g Log_10_ CFU^-1^ min^-1^). Concerning pH 8.0, whose R^2^ value was lower than 0.9, we could assume that the electrostatic repulsion between functional groups of the same charge determined that the value of the adsorption constant decreased 4.4 times more than for pH 3.0 and 4.6 times more than for pH 5.0 (Fig. 3B, Table 3).

**Table 3:** Values for the constants of the kinetic models used to fit the experimental data on the adsorption of PSB and orthophosphates on BC prepared at 500 °C 1 h.

Model for PSB						
pH ± 0.2	Pseudo-second-order			Elovich		
	*qe* Log_10_ CFU g^-1^	*k*^*2*^ g Log_10_ CFU^-1^ min^-1^	*R*^2^	α Log_10_ CFU g^-1^ min^-1^	β g Log_10_ CFU^-1^	*R*^2^
3.0	0.179	0.240	0.9991	31.3	0.040	0.9381
5.0	0.090	0.256	0.9792	64.1	0.020	0.9789
8.0	0.045	0.055	0.8587	67.3	0.009	0.8501

Model for orthophosphates						
pH ± 0.2	Pseudo-second order			Elovich		
	*qe* mg de P g^-1^	*k*^*2*^ g mg P^-1^ min^-1^	R^2^	α mg P g biochar^-1^ min^-1^	β g mg P^-1^	*R*^2^
3.0	− 0.797	0.279	0.9910	0.00006	0.009	0.1601
5.0	− 0.793	3.142	0.9902	− 0.066	0.021	0.0411
7.0	0.116	0.902	0.9286	3703	0.041	0.7700

Where *qe* is the amount of PSB or orthophosphates adsorbed at time *t* (PSB: Log_10_ UFC g^-1^) and (orthophosphates: mg g^-1^), *k*2 is the equilibrium rate constant of pseudo-second-order adsorption (PSB: g Log_10_ UFC^-1^ min^-1^) and (orthophosphates: g mg P^-1^ min^-1^), *α* is the initial adsorption rate (PSB: Log_10_ CFU g^-1^ min^-1^) and (mg P g biochar^-1^ min^-1^), *β* is the desorption constant (PBS: g Log_10_ CFU^-1^) and (g mg P^-1^) during any one experiment.

High *R*^2^ values result from the Elovich model, especially for pH 3.0 and 5.0 (0.9381 and 0.9789). The Elovich model has been used to describe the adsorption by chemical interactions between different compounds used as adsorbents^28,66^. According to the Elovich model, the adsorption rate (*α*) decreases with time due to the saturation of the material, which was tested at pH 3.0 and 5.0, obtaining values equal to 31.3 and 64.1 Log_10_ CFU min^−1^ respectively. Results showed that the cells adsorb faster at acidic pH than at alkaline pH and also that when saturation of the BC_500_ has reached velocity decreases. The desorption coefficient (β) was higher at acidic pH than at alkaline pH, showing that once BC_500_ saturation occurs, PSB desorption starts (Table 3).

The adsorption results for orthophosphates (Fig. 3C, D, Table 3), showed that at pH 7.0, phosphorus adsorption was low but occurred from the first minutes of contact, obtaining *qe* values equal to 0.116 mg g^−1^ BC (Fig. 3C). At pH 3.0 and 5.0, no adsorption occurred after 120 h (data not shown). At pH 7.0, a pseudo-second-order constant of 0.902 g mg P^−1^ min^−1^ and an R^2^ of 0.9286 resulted, and at pH 3.0 and 5.0, the experimental *qe* were negative, with − 0.797 mg g^−1^ BC and − 0.793 mg g^-1^ BC, respectively. Indicating that, at these pH values, BC has no ortho-phosphates adsorption capacity (Table 3). In the Elovich model applied at pH 7.0, the *R*^2^ value was 0.7700, and the *α* and *β* values were 3703 mg P g biochar^−1^ min^-1^ and 0.041 mg P^−1^, respectively.

### Co-inoculation of PSB to biochar and characterization

Table 2 shows the BC_500_/PSB characterisation; percentage of moisture increased (99 ± 2%) compared to BC_500_ without bacteria (3.6 ± 0.51%). Density (0.42 ± 0.1 ± 0.1 g cm^-3^), porosity percentage (64.3 ± 1.1%) and particle size did not change substantially with respect to BC_500_ (Table 2). The pH decreased with respect to BC_500_, obtaining a value of 6.4 ± 0.5; which could be related to the production of organic acids by the PSB^44^.

Proximate analysis showed an increase in the TOC percentage (54 ± 3%) observed in the co-inoculation of PSB compared to the percentage obtained in the uninoculated biochar. The above result could be related to the fact that the PSBs were grown in a medium rich in organic carbon and nitrogen. The bacterial biomass contains organic carbon and nitrogen. The sum of two carbon inputs could have led to the increase in the TOC percentage contributing to carbon concentration due to their organic nature and causing a decrease in fixed carbon of 3% regarding BC and also causing a slight increase in volatile carbon of 3.4% (Table 2). The ash percentage was similar in BC_500_/PSB and BC_500_ (2.0 ± 0.9%).

In the elemental analysis, the atomic percentage of carbon was 25.12%, oxygen increased from 26.9 to 73.61%, the hydrogen percentage was like BC_500_ (1.74%), and nitrogen decreased, obtaining a value of 0.04%. The atomic H/C ratio was for BC_500_ and BC_500_/PSB (0.02 and 0.05, respectively), which means that the addition of PSB did not change the highly condensed structure of BC_500_. On the contrary, the O/C and (O + N)/C atomic ratios increased when PSB was co-inoculated (2.930 and 2.931, respectively) suggesting that the addition of PSB in an aqueous solution increases the number of polar groups and the amount of interstitial water (Table 2).

As shown in Fig. 2E, F, the PSB were distributed on the surface of BC_500_ and formed a film. Adsorption of the bacteria increased by the acidic pH of BC_500_ (6.4 ± 0.5) and acidic pH of the PSB suspension used for co-inoculation of BC_500_ (1.0 × 10^7^ ± 1.0 × 10^1^ CFU mL^−1^ at pH 3.5). Regarding the morphology, only one type of cell was observed, characterised as short bacillus, part of the PSB consortium (*Serratia* sp., *Pseudomonas* sp., and *Kosakonia* sp.), which agreed with previous results^33,45^ (Fig. 2F).

### Evaluation of the effect of biochar co-inoculated with PSB (BC/PSB) on the growth of *Allium cepa* L., at a round plastic pot scale

**Figure 4:**
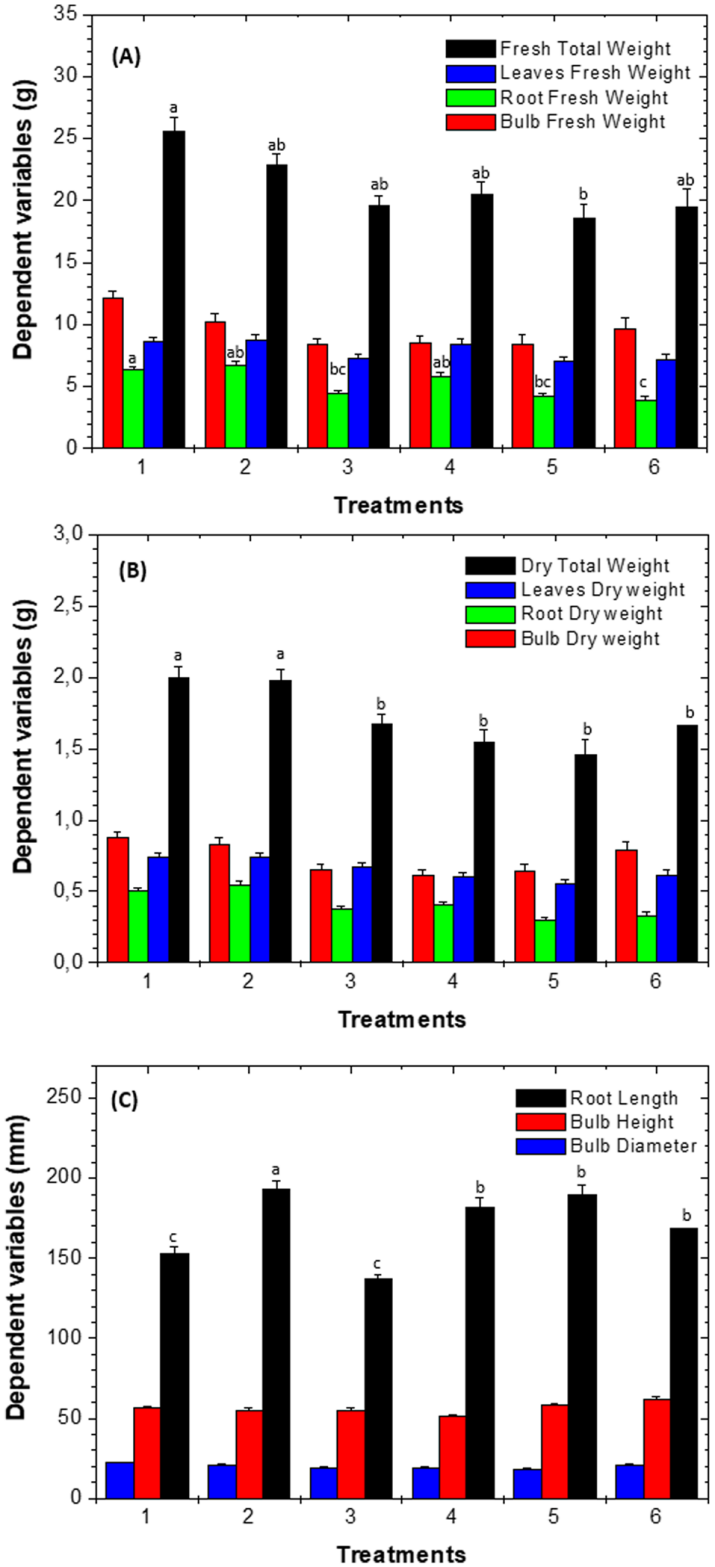
Effect of the biofertilizer on the growth of *A. cepa* L., in round plastics pots. (**A**) Variables for fresh-weights. (**B**) Variables for dry-weights. (**C**) Variables for the bulb. Figures letters represent the heterogeneous groups from Tukey's test and indicate significant differences between treatments (*p* < 0.05). T1: Abundagro + 5% Biochar + PSB; T2: Abundagro + 2% Biochar + PSB; T3: Abundagro + 5% Biochar alone; T4: Abundagro + 2% Biochar alone; T5: Abundagro; T6: Water.

Significant differences were observed (*p* < 0.05) in the total fresh weight of the plants between T1 (Abundagro + 5% Biochar + PSB) and T5 (Abundagro + 5% Biochar + PSB). T1 and T2, T3 and T4 had no statistical differences, T1 (Abundagro + 5% Biochar + PSB) plant weight was 25.57 ± 1.14 g, followed by T2, T4 and T3 with 22.80 ± 1.00, 20.50 ± 1.01 and 19.57 ± 0.82 g, respectively (Fig. 4A). In relation to root fresh weight (second variable in which significant differences were observed (p < 0.05), the highest values were observed in T2 (Abundagro + 2% Biochar + PSB) and T1 (Abundagro + 5% Biochar + PSB) with values of 6.68 ± 0.31 and 6.30 ± 0.23 g. No significant differences (*p* > 0.05) appeared between these two treatments (Fig. 4A). No significant differences (*p* > 0.05) were observed for bulb-fresh and leaf-fresh weight (Fig. 4A).

Figure 4B shows the plant’s total dry weight of leaves, roots, and bulbs. Significant differences were observed only for root dry weight (*p* < 0.05), obtaining the highest values for T2 and T1 with 0.54 ± 0.03 and 0.50 ± 0.02 g, respectively. No significant differences (*p* > 0.05) appeared among treatments regarding the variables of total dry weight, bulbs dry weight, and leaf dry weight (Fig. 4B).

When analysing the characteristics related to bulb height, bulb diameter, and root length, significant differences (*p* < 0.05) were observed only for root length, the best treatment was T2 with values of 193.50 ± 4.59 mm (Fig. 4C).

Although there were no significant differences between treatments (*p* > 0.05), the content of P, N, K, Ca y S in plants of T2 (Abundagro + 2% Biochar + PSB) is noteworthy (Table 4). Concerning the micronutrients, the differences were significant (*p* < 0.05) for Fe and Cu, for Fe the highest concentration was in T2 (0.1217 ± 0.0243 mg bulb^−1^), followed by T1 (0.0710 ± 0.0204 mg bulb^−1^) and T6 (0.0690 ± 0.0137 mg bulb^−1^), while the highest Cu content was found in T1 (0.0016 ± 0.0006 mg bulb^-1^), followed by T2, T3 and T4 (Table 4). No significant differences (*p* > 0.05) were observed for Na, Mn, and Zn. The values for T2 plants were 1.6455 ± 0.2156, 0.0262 ± 0.0038 and 0.0202 ± 0.0024 mg bulb^−1^, respectively. The B content ranged from 0.0194 ± 0.0023 to 0.0333 ± 0.0032 mg bulb^−1^ in all treatments (Table 4).

**Table 4:** Macronutrient and micronutrient content of *A. cepa* L., bulb.

Treatment	Treatment description	Macronutrient content (mg bulb^−1^) ^ns^					
		P	N	K	Ca	Mg	S
1	Abundagro + 5% Biochar + PSB	1.25 ± 0.13	10.87 ± 0.88	15.82 ± 1.88	10.04 ± 0.50	0.72 ± 0.11	1.85 ± 0.17
2	Abundagro + 2% Biochar + PSB	1.38 ± 0.14	12.21 ± 1.09	17.09 ± 1.23	12.46 ± 1.49	0.76 ± 0.07	2.09 ± 0.13
3	Abundagro + 5% Biochar alone	0.81 ± 0.10	7.17 ± 0.60	10.16 ± 0.99	7.56 ± 0.81	0.49 ± 0.06	1.27 ± 0.12
4	Abundagro + 2% Biochar alone	1.02 ± 0.29	8.79 ± 1.87	14.70 ± 3.45	10.33 ± 2.10	0.58 ± 0.15	1.51 ± 0.41
5	Abundagro	0.76 ± 0.14	7.58 ± 0.73	10.44 ± 0.93	7.81 ± 0.74	0.49 ± 0.03	1.26 ± 0.18
6	Water	1.17 ± 0.22	11.08 ± 2.17	16.59 ± 3.35	12.20 ± 2.90	0.90 ± 0.31	1.88 ± 0.31
	CV (%)	33.92	30.48	32.28	35.45	48.72	31.02

Treatment	Treatment description	Macronutrient content (mg bulb^−1^)					
		Na^ns^	Fe	Mn^ns^	Cu	Zn^ns^	B^ns^
1	Abundagro + 5% Biochar + PSB	1.2678 ± 0.0761	0.0710 ± 0.0204 ab	0.0258 ± 0.0079	0.0016 ± 0.0006 a	0.0182 ± 0.0026	0.0291 ± 0.0016
2	Abundagro + 2% Biochar + PSB	1.6455 ± 0.2156	0.1217 ± 0.0243 a	0.0262 ± 0.0038	0.0006 ± 0.0004 ab	0.0202 ± 0.0024	0.0333 ± 0.0032
3	Abundagro + 5% Biochar alone	0.9166 ± 0.0918	0.0490 ± 0.0110 b	0.0176 ± 0.0030	0.0008 ± 0.0004 ab	0.0148 ± 0.0033	0.0199 ± 0.0013
4	Abundagro + 2% Biochar alone	1.4225 ± 0.4615	0.0435 ± 0.0073 b	0.0157 ± 0.0032	0.0005 ± 0.0004 ab	0.0144 ± 0.0033	0.0229 ± 0.0048
5	Abundagro	0.9165 ± 0.0758	0.0416 ± 0.0028 b	0.0139 ± 0.0019	0.0003 ± 0.0002 b	0.0119 ± 0.0013	0.0194 ± 0.0023
6	Water	1.1793 ± 0.1682	0.0690 ± 0.0137 ab	0.0139 ± 0.0023	0.0004 ± 0.0001 b	0.0157 ± 0.0018	0.0343 ± 0.0036
	CV (%)	39.04	46.25	35.72	71.04	32.87	24.98

Letters show heterogeneous groups based on Tukey's test and indicate significant differences among treatments (*p* < 0.05). ns = no significant difference (*p* > 0.05). CV = coefficient of variation.

### Effect of biofertiliser on nutrient content in the soil

When analysing the variation in soil fertility before and after sowing (Supplementary Material S1), significant differences result for most variables (Table 5, Supplementary Material S3).

**Table 5:** Variation of physical and chemical characteristics of soil used (initial-final values) before and after *A. cepa* L. cultivation in pots.

Treatment	Treatment description	P tot (mg Kg^−1^)	P ext (mg Kg^−1^)	P sol (mg Kg^−1^)	CEC	EC
1	Abundagro + 5% Biochar + PSB	309.08 ± 51.98^b^	57.80 ± 4.95^b^	6.57 ± 0.18^b^	− 0.58 ± 0.47^c^	0.33 ± 0.03^a^
2	Abundagro + 2% Biochar + PSB	315.87 ± 51.35^b^	67.60 ± 3.26^ab^	6.61 ± 0.37^b^	2.03 ± 0.42^b^	0.16 ± 0.07^ab^
3	Abundagro + 5% Biochar alone	454.80 ± 73.23^ab^	60.03 ± 2.38^b^	5.19 ± 0.45^bc^	0.83 ± 0.42^bc^	0.32 ± 0.03^ab^
4	Abundagro + 2% Biochar alone	748.43 ± 68.30^a^	82.43 ± 2.87^a^	8.50 ± 0.25^a^	5.43 ± 0.60^a^	0.22 ± 0.05^ab^
5	Abundagro	453.72 ± 64.29^ab^	77.93 ± 4.38^a^	4.65 ± 0.31^c^	1.63 ± 0.57^bc^	0.27 ± 0.03^b^
6	Water	201.95 ± 104.20^b^	74.93 ± 4.31^ab^	9.60 ± 0.29^a^	1.43 ± 0.44^bc^	0.33 ± 0.03^a^
	CV (%)	45.92	14.20	27.71	111.37	− 25.85

Treatment	Treatment description	OC (%)^ns^	OM (%)^ns^	N (%)^ns^	K ext (mg Kg^−1^)^ns^	Na ext (mg Kg^−1^)
1	Abundagro + 5% Biochar + PSB	− 2.17 ± 0.14	− 3.74 ± 0.23	− 0.18 ± 0.01	− 48.33 ± 13.76	97.33 ± 5.63^ab^
2	Abundagro + 2% Biochar + PSB	− 0.81 ± 0.42	− 1.39 ± 0.72	− 0.07 ± 0.03	− 10.00 ± 20.33	79.33 ± 6.63^b^
3	Abundagro + 5% Biochar alone	− 1.42 ± 0.91	− 2.35 ± 1.61	− 0.11 ± 0.08	− 28.33 ± 20.88	105.00 ± 3.21^ab^
4	Abundagro + 2% Biochar alone	− 1.81 ± 0.39	− 3.11 ± 0.67	− 0.15 ± 0.03	15.00 ± 21.72	78.67 ± 10.39^b^
5	Abundagro	− 1.10 ± 0.22	− 1.92 ± 0.38	− 0.09 ± 0.02	− 33.33 ± 19.61	118.00 ± 4.47^a^
6	Water	− 1.38 ± 0.19	− 2.27 ± 0.23	− 0.12 ± 0.02	11.67 ± 24.14	83.67 ± 5.90^ab^
	CV (%)	− 33.72	− 34.25	− 33.86	− 164.13	16.98

Treatment	Treatment description	Ca (mg Kg^−1^)	Mg (mg Kg^−1^)	Fe (mg Kg^−1^)	B (mg Kg^−1^)	Mn (mg Kg^−1^)
1	Abundagro + 5% Biochar + PSB	1313.33 ± 93.33^c^	16.83 ± 1.76^b^	5.92 ± 0.06^b^	0.10 ± 0.03^b^	41.77 ± 1.61^ab^
2	Abundagro + 2% Biochar + PSB	1810.00 ± 88.51^ab^	5.50 ± 3.73^c^	2.55 ± 0.05^e^	0.08 ± 0.03^b^	16.68 ± 2.12^c^
3	Abundagro + 5% Biochar alone	1548.33 ± 87.99^abc^	30.17 ± 1.25^a^	3.24 ± 0.01^d^	0.14 ± 0.03^ab^	36.08 ± 1.35^ab^
4	Abundagro + 2% Biochar alone	1863.33 ± 115.06^a^	13.50 ± 1.52^bc^	3.58 ± 0.01^c^	0.12 ± 0.03^b^	35.13 ± 1.71^ab^
5	Abundagro	1481.67 ± 103.42^abc^	18.33 ± 1.36^b^	1.68 ± 0.07^f^	0.05 ± 0.02^b^	29.45 ± 1.89^bc^
6	Water	1375.00 ± 98.65^bc^	5.50 ± 2.36^c^	8.27 ± 0.01^a^	0.24 ± 0.01^a^	45.02 ± 4.28^a^
	CV (%)	14.45	61.74	58.17	53.35	29.62

Treatment	Treatment description	Cu ext (mg Kg^−1^)	Zn ext (mg Kg^−1^)	S ext (mg Kg^−1^)	Ca/Mg	Ca/K^ns^
1	Abundagro + 5% Biochar + PSB	0.31 ± 0.02^b^	2.64 ± 0.52^a^	82.68 ± 4.99^a^	2.63 ± 0.67^bc^	7.07 ± 0.56
2	Abundagro + 2% Biochar + PSB	0.14 ± 0.04^bc^	− 1.61 ± 0.24^c^	45.95 ± 6.17^bc^	7.05 ± 0.64^a^	7.23 ± 1.09
3	Abundagro + 5% Biochar alone	0.17 ± 0.02^bc^	− 0.90 ± 0.29^c^	63.03 ± 3.39^ab^	0.87 ± 0.64^c^	6.67 ± 0.87
4	Abundagro + 2% Biochar alone	0.21 ± 0.02^bc^	1.18 ± 0.19^ab^	55.63 ± 6.99^ab^	5.57 ± 0.52^ab^	6.48 ± 1.00
5	Abundagro	0.08 ± 0.02^c^	− 0.73 ± 0.54^bc^	65.70 ± 6.08^ab^	3.08 ± 0.37^bc^	7.33 ± 0.64
6	Water	1.40 ± 0.04^a^	1.94 ± 0.29^a^	19.50 ± 8.02^c^	5.65 ± 0.78^ab^	4.65 ± 1.28
	CV (%)	130.55	410.54	38.59	55.99	15.17

Treatment	Treatment description	Mg/K	Ca Mg/K ^ns^	Sat Mg	Sat K^ns^	Sat Na
1	Abundagro + 5% Biochar + PSB	0.15 ± 0.04^ab^	7.32 ± 0.57	− 0.31 ± 0.09 ^ab^	− 1.24 ± 0.13	0.66 ± 0.08^ab^
2	Abundagro + 2% Biochar + PSB	− 0.74 ± 0.04^c^	7.28 ± 1.12	− 0.77 ± 0.09 _c_	− 1.15 ± 0.23	0.19 ± 0.11^b^
3	Abundagro + 5% Biochar alone	0.26 ± 0.05^a^	6.85 ± 0.90	− 0.09 ± 0.07 _a_	− 1.4 ± 0.21	0.64 ± 0.06^ab^
4	Abundagro + 2% Biochar alone	0.06 ± 0.04^b^	6.47 ± 1.04	− 0.60 ± 0.06 _bc_	− 1.02 ± 0.19	0.19 ± 0.14^b^
5	Abundagro	0.17 ± 0.02^ab^	7.48 ± 0.65	− 0.31 ± 0.04 ^ab^	− 1.16 ± 0.13	0.85 ± 0.05^a^
6	Water	0.01 ± 0.04^b^	4.65 ± 1.35	− 0.53 ± 0.08 _bc_	− 0.80 ± 0.25	0.46 ± 0.09 ^ab^
	CV (%)	− 2591.07	15.87	− 56.04	− 15.07	53.91

Treatment	Treatment description	Sat Ca ^ns^	Sat Hum			
1	Abundagro + 5% Biochar + PSB	0.93 ± 0.16	6.53 ± 0.54^b^			
2	Abundagro + 2% Biochar + PSB	1.62 ± 0.35	9.12 ± 0.56^a^			
3	Abundagro + 5% Biochar alone	0.60 ± 0.28	8.82 ± 0.49^a^			
4	Abundagro + 2% Biochar alone	1.20 ± 0.12	9.12 ± 0.45^a^			
5	Abundagro	0.72 ± 0.12	8.98 ± 0.12^a^			
6	Water	0.97 ± 0.36	7.72 ± 0.37^ab^			
	CV (%)	36.29	12.50			

Letters show heterogeneous groups based in Tukey's test and indicate significant differences among treatments (*p* < 0.05). ns = no significant differences (*p* > 0.05). CV = coefficient of variation.

The pH of T1 and T3 increased by 0.25 ± 0.01 and 0.29 ± 0.02 units compared to the initial pH (7.69 ± 0.04 and 7.70 ± 0.05). These two treatments contained 5% (w/w) BC, but T1 was the biofertiliser containing organic acid-producing PSB generating slight acidification of the soil, which was observed in the less alkaline pH of T1 compared to T2.

In all treatments, the EC value decreased at the end of the trial (Supplementary Material S3), the variation was higher in T1 and T6 (Table 5). The final EC for the six treatments ranged between 29.1 ± 1.46 and 30.2 ± 1.39 meq 100 g^−1^, with the lower variation occurring in T1 (− 0.58 ± 0.47 meq 100 g^−1^), suggesting a higher concentration of exchange complex bases in the soil solution, mainly Ca, Mg, Na, and K. Low variations in Ca, Mg, and Na concentrations occurred, indicating a greater plant nutrient assimilation in conditions where nutrient leaching was absent for any of the treatments (Table 5).

The Ca/Mg ratio values ranged from 24.7 to 27.7, indicating Mg deficiency and that variation occurred in T2 with differences between treatments. The Mg/K ratio ranged from 0.7 to 0.8, indicating Mg deficiency and a remarkable variation in T3 (Table 5).

The difference in the total P content (TP) in the soil was lower in T1, T2 and T6, indicating a high mobilisation of this element in these three treatments. Additionally, it was remarkable the total P concentration reached in T1, T2, T3 and T4 (1464 ± 127, 1523 ± 126, 1469 ± 179 and 1445 ± 167 mg Kg^-1^), which was associated with the BC containing. The soluble P content of recently produced BC was 46.3 mg L^−1^ reported in the complete nutritional analysis and or PSB cultivated in MT11B medium whose formulation has phosphate rock (PR) as a P source. The PS content in the biofertiliser was 21.9 mg L^−1^ (Table 5).

Extractable P in the soil is associated with Ca and Mg when the soil pH is alkaline, as was the case in this study. The low variation in the concentration of extractable P in soil occurred in T1, T2 and T3 (variations: 57.80 ± 4.95, 67.60 ± 3.26 and 60.03 ± 2.38 mg Kg^−1^ respectively) with final concentrations of 101.2 ± 12.1, 93.4 ± 8.0 and 88.0 ± 5.8 mg Kg^−1^ for T1, T2 and T3 respectively. Treatments T4, T5 and T6 showed the highest variation in the final concentration of extractable P compared to treatments T1, T2 and T3. In T1 and T2, it was attributed to the presence of PSB and self-release of organic acids able to solubilise P from the extractable P fraction (Table 5 and S3).

As for soil soluble P, the concentration in the soil of T1 was one of the lowest (6.43 ± 0.45 mg Kg^−1^) compared to the other treatments and is related to the P content in the bulbs of the same treatment (T1), where one of the highest P contents found in the onion bulbs with 1.25 ± 0.13 mg bulb^−1^ (Table 4). Total fresh weight (25.57 ± 1.14 g) and total dry weight (2.00 ± 0.08 g) of onions were the highest of all treatments in T1 with significant differences in TFW (*p* < 0.05) (Fig. 4).

Soluble P showed the highest variability at the control, but lower assimilation in *A. cepa* L., bulb due to possible precipitation with Ca or Mg. T4 with BC 2% showed high Ca content and high total P, high available P, and high P in solution, but the P content in the bulb was lower than in the other treatments due to possible precipitation with Ca.

The treatments that had the great variation in moisture saturation were due to the soil retaining the least amount of water, which occurred in most of the treatments except for T1, where moisture saturation had the lower variation, indicating that this soil had the best water retention properties due to the concentration of biofertiliser used for formulation (Table 5).

## Discussion

### Characterisation of Caribbean pine sawdust and BC_500_

The physical and chemical characteristics of the SCP used in this study were like those reported by other authors who have produced biochar from pine sawdust and are consistent with the high contents of structural polymers (Supplementary Material S3)^9,20,21^. The high C/N ratio depends on the higher amounts of slowly degrading carbon and low nitrogen contents. Part of this carbon comes from the phenyl propane monomers that constitute the lignin polymer (p-coumaryl alcohol, coniferyl alcohol and sinapyl alcohol)^67^. These monomers links by ether, C–C, aryl-C bonds and have different functional groups such as methoxy, hydroxy and carbonyl^22^. These characteristics give lignin a high degree of aromaticity and hydrophobic characteristics, which are decisive in the final structure of the biochar^9^. On the other hand, the crystalline structure and hydrogen bonds present in cellulose also contribute to increasing the stability of SCP^20,67^. Those are reasons why SCP is considered an excellent feedstock for biochar production and is an alternative use of lignocellulosic biomass produced by the forestry sector worldwide^9,49^.

During pyrolysis, three stages occur. The first was the dehydration of the SCP that occurred at temperatures between 50 and 300 °C^27^. In the second stage, combustion occurred under reduced oxygen conditions, causing the labile fractions of C, N and S to volatilise (approximately 250 and 350 °C). Likewise, hemicellulose and cellulose undergo thermal degradation processes, and graphene nuclei begin to form^27,28,49^. On the other hand, the decomposition and reorganisation of lignin by-products start at higher temperatures (350 and 450 °C)^22^. The most-reported are p-hydroxyphenyl, guaiacyl and syringyl derivatives^9^. These intermediates are considered responsible for contributing to the fraction of carbon to be transformed into condensed biochar structures^9,21^. Finally, in the third stage, carbonisation and condensation processes are carried out to generate the most stable fraction of BC, which starts at temperatures of 400 °C and reaches 500 °C^19,22,28^.

As shown in Table 2, the moisture content of BC_500_ decreased (3.6 ± 0.51%) due to the dehydration process of the CPS (7.0 ± 0.31%). The increases in density (0.42 ± 0.1 ± 0.1 g cm^-3^) and porosity percentage (64.3 ± 1.1%) occurred by the carbonisation and condensation processes of the BC at 500 ºC. On the other hand, the rearrangement of condensed carbon in the BC_500_ matrix generated a decrease in particle size (3.0 ± 0.7 mm) (Table 2, Fig. 2C, D). A similar result associated with changes in porosity, particle size, and surface area has been previously published^49^. Some authors have found that when increasing temperature, porosity also increases because of the removal of volatile matter, leading to an increase in surface area when using pine sawdust^49^. On the other hand, at temperatures ranging between 300 and 400 °C, tars are formed that, when volatilised at temperatures of 500 °C, leave irregular pore-like spaces ^23^.

Concerning pH, an increase from 3.7 ± 0.08 (CPS) to 7.1 ± 0.6 (BC500) occurred, compared to the temperature and the initial CPS composition. As presented in Supplementary material S2, the CPS has several elements, such as Calcium, Sodium, Potassium, Manganese, Aluminium, among others. The heat treatment produced ashes that concentrated the alkaline minerals and favoured the loss by volatilisation of acidic functional groups (phenolic groups and carboxylic acids)^12,19,24^.

Proximate analysis for the CPS shows that being the unique material that underwent a drying process, it is feasible that it has low CF contents while the highest proportion of carbon is associated with the volatile fraction (Table 2). Chang et al.^9^ and De Bhowmick et al.^27^ (2018) observed that raw pine sawdust tends to have a higher percentage of VC independent of the pine species used; which is consistent with our results as Caribbean pine sawdust was used^9,27^.

In the proximate analysis for the BC, an 11.85% increase in CF (26 ± 2%) compared to CPS (14.15 ± 0.21%) and a decrease in CV (71.6 ± 2.4%) occurred. These results suggest the feasible production of a BC_500_ with condensed carbon, and volatilisation losses of H_2_O, CO_2_, CO, CH_4_, H_2_ and S decreased when produced under reduced oxygen conditions^68,69^.

Another factor that helped the CF increase was the high CPS lignin content (40.9%) because lignin is carbonised, reorganised and condenses at temperatures between 450 and 500 °C to give structure to the biochar^21^. Similar results have been commented by Chang et al.^9^; Kan et al.^20^, Igalavithana et al.^23^ and Lehmann et al.^70^ in their studies produced biochar from pine sawdust and observed that at temperatures between 500 and 600 °C fixed carbon increases at the expense of lignin content^9,20,23,70^.

Other results that helped support the successful production of BC_500_ were those obtained from elemental analysis and atomic ratios. Table 2 shows that carbon concentration increased (71.04%), while oxygen and hydrogen decreased compared to carbon (26.9 and 1.74%). These proportions changed due to cellulose, hemicellulose, and lignin depolymerisation. On the other side, carbon, hydrogen and oxygen proportions also changed due to the amorphous carbon matrix produced and the possible formation of polyaromatic graphene^24^. Additionally, N was more concentrated in BC_500_ (0.32%) compared to CPS (0.08%), which could be related to inorganic forms of nitrogen such as NO_3_ and NO_2_, which can result from thermal oxidation of part of the total nitrogen and ammonium^49^. Haddad et al.^19^ reported that in pine sawdust biochar and impregnated with inorganic salts, the elemental nitrogen content can be increased up to 0.3%^19^.

Regarding the atomic ratios, the decrease relates to the effect of the heat treatment of 500 °C for one h, which has been commented on by different authors who have worked with timber tree by-products^9,22,33,71^. Like these authors in this work, the decrease of H/C from 0.12 (CPS) to 0.02 (BC) was due to the removal of water, OH and H through dehydration and dehydrogenation processes^27,28^. The value obtained indicated an increase in aromaticity of BC_500_ and possibly has graphite structures because it was lower than 0.1^68^. The decrease in O/C ratio from 0.95 (CPS) to 0.38 (BC_500_) suggests that BC_500_ is mature, stable and less polar, due to the loss of OH groups and aliphatic groups^72,73^. Changes in the (O + N)/C ratio from 0.954 in CPS to 0.383 in BC_500_ suggested a decrease in the amount of oxygen- and hydrogen-bearing functional groups associated with less polar biochar (Table 2). The results of the atomic O/C and H/C ratios were less than 0.45 and 0.6, respectively, indicating that the BC_500_ produced in this study could be used in agriculture because of its high stability and would help to improve the physical, chemical, and biological properties of soil^12,19,33^.

### Infrared spectroscopy with Fourier arrays

The presence of symmetrical C–O single bonds (1025 cm^-1^), C=C double bonds (1585 cm^-1^) and C=O double bonds (1740 cm^-1^) are characteristic in sawmill by-products containing cellulose, hemicellulose and lignin^9^. The presence of O–H stretches between 3200 and 3500 cm^−1^ was not distinct. Only an expected small band appear at 3330 cm^-1^ to be more noticeable as it is related to the abundance of hydrogen-bonded OH groups; interstitial water located between cellulose and hemicellulose^49^. Its low intensity could be related to the pre-drying process done to the SCP before producing BC_500_ (Fig. 1). In BC_500_, the signals associated with the most labile carbon (C–O) and hydroxyl groups (O–H) disappeared; this proved the removal of water, aliphatic and polar groups at 500 ºC. A similar result has been reported by Lou et al.^49^; in their study, they produced biochar at different temperatures using sawdust as raw material and observed that biochar performed at 500 °C lost more O–H and aliphatic functional groups than those obtained at 300 °C^49^.

On the other hand, most stable bonds such as C=C (1585 cm^-1^), C=O double bonds (1740 cm^−1^) and C–C bonds (1365 cm^−1^) were concentrated at BC_500_ and were possibly responsible for decreasing the polarity (Fig. 1). These results were related to the H/C and O/C aromaticity index, obtaining values below 0.3. These results are like those obtained by several authors^19,22,28,49^.

### Adsorption studies for PSB

The bacterial wall consists of polysaccharides, proteins and lipids containing amino, carboxyl, phosphate and sulphate groups that can act as polyelectrolytes^45^. The PSB adsorption to BC_500_ depends on the pH of the solution and the initial PSB concentration^25,26,74^. At acidic pH (3.5) lower than pHzpc (4.1), BC_500_ becomes positively charged, favouring the attraction of bacteria having functional groups negatively charged^33^. Other factors that enabled the adsorption of PSBs were the porosity of BC_500_, the contact surface and the production of exopolysaccharides, which are also negatively charged^25^.

On the contrary, at pH 5.0 and 8.0, the BC_500_ changes its surface charge and increases the negative charges (pH > pHzp), causing an electrostatic repulsion between the PSB and the BC_500_. This electrostatic repulsion was more evident at pH 8.0 than at pH 5.0, indicating that at pH 5.0 coexist both positively and negatively charged functional groups^25^.

### Adsorption studies for orthophosphates

According to the adsorption studies results, BC_500_ has a low adsorption capacity for orthophosphates (Fig. 3, Table 3)**.** Only a low quantity of orthophosphates (0.116 mg g^-1^) adsorbed at pH 7.0. It is possible that at pH 7.0 and 5.0, part of the BC_500_ surface becomes negatively charged (pH > pHzp) and generates an electrostatic repulsion with the orthophosphate ions, which are also negatively charged^49^. A similar result reported by Lou et al*.*^49^, in their work produced biochar at 300 and 600 °C, observing that phosphorus removal was low at pH under the isoelectric point^49^. On the other hand, the graphene layers formed during the pyrolysis process can also acquire a negative charge at pH above pHzpc, contributing to a decrease in the adsorption of ions negatively charges^75^.

### Formulation of PSB to biochar and characterization

A wide variety of materials served for the co-inoculation of PSB on solid carriers; these must guarantee the survival of the microorganisms, their biological activity and protect them from biotic and abiotic stresses^25^. An appropriate carrier should be inexpensive, easy to use, easy to acquire, allow gas exchange and maintain moisture. On the other hand, it must be biocompatible for microorganisms and plants^26,34,42^.

In BC_500_/PSB, some changes in the chemical characteristics of the material occurred, being the most notorious the increase in VC (75 ± 3%), the elemental content of oxygen (73.61%), hydrogen (1.23%), atomic ratio H/C (0.05) and O/C (2.93), (Table 2). These were related to the hydration of BC_500_ when co-inoculated with a PSB suspension containing water, carbon, nitrogen, and other elements provided by the culture medium used to culture PSB. Under the experimental conditions, two stages could occur during the co-inoculation. In the first one, the PSB adsorb to the surface of BC_500_, favoured by the pH of the PSB suspension (3.5), this pH value is below zero charge point of BC_500_ and determined that the surface acquires a positive charge, allowing the PSB to adsorb through chemical interactions (Table 2, Fig. 3). Subsequently, the PSB were able to form a biofilm by the production of exopolysaccharides, and the adhesion to the BC_500_ was more stable. Finally, the spatial distribution of the PSB was not only superficial but also distributed in and around the pores (Fig. 4)^33^.

### Evaluation of biochar co-inoculated with PSB effect on the growth of *Allium cepa* L., cultivated at nursery scale

Fertilisation of onion crops is carried out without a prior comprehensive soil diagnosis, based on an empirical application of solid fertilisers of inorganic origin^76^. Álvarez-Hernández et al.^76^ and Blanco & Lagos^77^, reported that P extraction per productive cycle is 35 ± 5 kg ha^−1^^76,77^. When phosphate fertilisers are in excessive amounts, they cause P fixation, characterised by the presence of high concentrations of P in the form of secondary phosphate minerals^78^. Therefore, fertilisation should be associated with a comprehensive and balanced application of the elements needed by the plant^79^. The soil to evaluate the effect of the biofertiliser on round plastic pots was selected, as previously mentioned, according to the extractable P content (168 mg Kg^-1^) and pH (7.73) reported in the nutritional analysis before sowing (Supplementary material S1).

Biofertilizer and BC concentrations evaluated in this study come from a previous study^33^, where three concentrations of a BC-based bioproduct containing Caribbean pine sawdust and PSB (*Pseudomonas* sp., *Serratia* sp., and *Kosakonia* sp.) were evaluated on the growth of *A. cepa* L., at concentrations of 1, 2 and 5% of the bioproduct. In this study, the dry weights (mg) and heights (cm) of the onions were significantly different (*p* < 0.05) when the 2 and 5% bioproduct were employed^33^. The BC properties as the microbial carrier are supported by the high PSB populations, biofilm arrangement of microorganisms in the bioproduct, and recovery of bacteria from the substrate used for planting in all these treatments that assayed the effect of the bioproduct. For all these reasons, we studied two biofertiliser concentrations (2 and 5%) on the growth of *A. cepa* L., in round plastic pots.

During the last few years, the use of bioinoculants to improve crop yields and nutrient uptake efficiency has become crucial to achieving field efficacy and commercial success. Therefore, must be reached the product quality and stability required by the market. Two main aspects for the development of successful inoculants at the crop level in field plots are the selection of the strain or strain mixture and a proper formulation, including the carrier of the bacteria^78,80–82^. The present study addresses the two aspects mentioned by evaluating the biofertiliser on the growth of *A. cepa* L., in round plastic pots, using agricultural soil from the Department of Boyacá where this plant species is grown.

The onion plants were harvested four months after transplanting. Table 4, shows the concentration of nitrogen, phosphorus, and potassium in the bulbs (mg bulb^-1^) at the end of the experiment. The application of PSB to the plants treated with Abundagro and Biochar favoured the uptake of these nutrients in the trial plants. PSBs have been reported as mutualistic symbiotic microorganisms, which colonise the roots of most crop plants and can enhance nutrient mobility and uptake at the plant level^78,83,84^. Rafique et al.^37^ report that the abundance of microorganisms can increase in BC amended soils due to the porous structure of the material which can be a potential habitat for bacteria, increasing the surface area for nutrient uptake^37^. Some studies have shown positive effects of BC addition on plant growth; however, plant responses vary depending on the soil. On soils with high nutrient content, there may not be significant benefits in terms of plant biomass gained in a single growing season^35,74,85^.

Soil inoculation with PSB-containing biofertilisers promotes plant growth^34^. In the present study, this effect was evident in the total dry weight (TDW) (g), fresh weight and root length of the plants that received the biofertilizer (Fig. 4). This may be attributed to the increase in the availability of nutrients such as N and P, a consequence of the modification of the presence and concentration of organic compounds in the rhizosphere, which together with the increase in root area produced in response to the presence of plant growth regulators such as indole acetic acid (IAA), favours the uptake of available forms of these elements^34,78,86^.

Balemi et al.^87^ reported a significant increase in the percentage of nutrients in the bulb of *A. cepa* L., especially N, as a result of inoculation with *Azotobacter* sp., a bacterial strain producing growth regulators that enhanced root development, leading to increased nutrient uptake^87^. In the present study, PSB inoculation favoured bulb N content in plants treated with Abundagro and Biochar 2% (w/w), treatment with the highest values for root length.

In the present study, PSB inoculation favoured bulb N content in plants treated with Abundagro and Biochar 2% (w/w), treatment with the highest values for root length. Balemi et al.^87^ also reported a significant increase in the percentage of this nutrient in the bulb of *A. cepa* L., resulting from inoculation with *Azotobacter* sp., a bacterial strain producing growth regulators that enhanced root development, leading to increased nutrient uptake. Despite that variation of ammonium and nitrates may be associated with the use of chemical fertilisers which are rotated in crops^88^, the low and medium contents of K and B required for the growth of *A. cepa* L., the roots as the first point of contact between the BC particles and the growing plants^89^ favour the absorption of the nutrients available in this organic material.

Besides root length, root weight and total fresh weight of plants were higher in the treatments with biofertiliser (Fig. 4) as reported by Rafique et al.^34^, which demonstrated that the inoculation with PSB improved the growth of roots and maize plant shoots^34^. In the present work, the addition of BC and the application of PSB favoured the described effect by increasing the biomass (T1) and root length (T2) of the inoculated plants (Fig. 4).

The use of the biofertilisers in T1 (5% biofertiliser) increased cation exchange capacity (CEC) (29.6 ± 1.14) and pH (7.6 ± 0.04) (Table 5 and S3). These can be due to ashes accumulation, which is generally composed of alkali (–Na, –K) and alkaline earth (–Mg, –Ca) metal carbonates, phosphates and small amounts of organic and inorganic compounds^90^. When the pH presents values around neutrality, the positive charge of Fe and Al oxides thus decreases the affinity for P. Additionally, increasing soil pH due to an increase in alkali metal oxides (Ca, Mg and K) can decrease the solubility of reactive Al and thus reduce P immobilisation^91^ improving the growth of onion at pH close to neutrality as reported by Tekeste et al.^92^.

Hardie et al.^93^ suggest that changes in P availability and leaching after BC application to soil result from a combination of physicochemical mechanisms, such as modification of soil pH, formation of metal phosphate complexes, microbial activity promotion, and increased the mineralisation of phosphate^93^.

Concerning other macronutrients such as N and K, an increase in the final soil concentration was observed, evidenced by the negative sign in the variation of the concentrations reported in Table 5. Rehman et al.^94^ showed that soil extractable P^3^ and other macronutrients increased before and after wheat harvest with BC application and that the response depends on soil type, cultivation time and the concentration of biochar formulated in the soil^94^.

Post-harvest soil analysis showed the effect of treatments on pH. More pH variation with a tendency towards alkalinity occurred in both the biofertiliser and BC treatments. Rehman et al.^94^ showed, as in the present study, that BC slightly changes soil pH with a tendency towards alkalinity^94^. Borno et al.^91^ mentioned that base saturation is positively related to soil pH because a high base saturation value would indicate that the exchange sites of a soil particle are coupled with non-acidic ions^91^. Similar results we obtained in this study. Table 5 shows negative Mg and Ca saturation values in all treatments, indicating an increase of these elements at the end of the biofertiliser evaluation in round plastic pots. While for Na and Ca there was no increase of these bases in the soil analysed after harvest. Mukhtar et al.^81^ also documented that the combined use of inorganic phosphate, bacterial strains and some carrier materials are beneficial in improving soil pH and root surface area through increased root proliferation^81^.

## Conclusions

The effects of the biofertiliser were related to increased germination, seedling growth, nutrient assimilation, and plant growth because PSB immobilised in BC promoted the mobilisation of nutrients, particularly P, during the cultivation of *Allium cepa* L. The above was demonstrated in T1 and T2, in which the bioinoculant (BC_500_/PSB) favoured total P concentrations of 1.25 ± 0.13 and 1.38 ± 0.14 mg bulb^–1^, respectively. These are the highest contents compared to the other treatments evaluated.

These results at the greenhouse scale should be validated in the field, taking the crop to harvest time to verify the effect of the product and the efficiency of nutrient use as part of the development of biological inputs for the agricultural sector. This becomes relevant if we consider that soils with onion crops have received mineral fertilisers for a long time and therefore have high total phosphorus content but moderate or low concentrations of available forms of the nutrient.

The bio-inoculant obtained in this study from biochar combined with autochthonous PSB is an effective strategy to release the nutrient from these unavailable forms and increase the contents of P ext and P sol. Our bio-inoculant after the several crop cycles in soils (with high amounts of nutrients) can improve the availability of the nutrients, which will make it possible to decrease future doses of phosphate mineral fertilisers and have a positive impact on the exploitation of the phosphate rocks from which they are.

This work presents the benefits of a new product based on bacteria naturally associated with onion and an organic material (BC_500_) which, in addition to serving as a bacterial carrier, has effects on increasing the adsorption area of highly reactive nutrients, reducing their leaching or precipitation with other nutrients and fixation to the solid matrix of the soil.

## Supplementary Information

Supplementary Information.

## Data Availability

Major datasets generated and analysed during the current study are available in the [Figshare] repository, [https://doi.org/10.6084/m9.figshare.20060213.v1]. Also, part of datasets generated and analysed during the current study are included in this published article [and its supplementary information files].
