# Supplementary material for: Production of pine sawdust biochar supporting phosphate-solubilizing bacteria as an alternative bioinoculant in Allium cepa L., culture
Source: Sci Rep. 2022 Jul 27;12:12815. doi: 10.1038/s41598-022-17106-1 (PMC9329452; doi:10.1038/s41598-022-17106-1)
Supplement: Supplementary file 1 — Supplementary Information. [file 41598_2022_17106_MOESM1_ESM.docx]

**Production of pine sawdust biochar supporting phosphate-solubilizing bacteria as an alternative bioinoculant in *Allium cepa* L., culture**

Andrea Blanco-Vargas ^1,2,3^**^†^**, María A. Chacón-Buitrago ^1^, María C. Quintero-Duque ^1^, Raúl A. Poutou-Piñales ^2^, Lucia A. Díaz-Ariza ^3^, Carlos A. Devia-Castillo ^4^, Laura C. Castillo-Carvajal ^5^, Daniel Toledo-Aranda ^5^, Christiano da Conceição de Matos ^6^, Wilmar Olaya-González ^3^, Oswaldo Ramos-Monroy ^7^, Aura M. Pedroza-Rodríguez ^1*^

^1^ Laboratorio de Microbiología Ambiental y de Suelos, Unidad de Investigaciones Agropecuarias (UNIDIA). Departamento de Microbiología. Facultad de Ciencias. Pontificia Universidad Javeriana. Bogotá, D.C., Colombia.

^2^ Laboratorio de Biotecnología Molecular, Grupo de Biotecnología Ambiental e Industrial (GBAI). Departamento de Microbiología. Facultad de Ciencias. Pontificia Universidad Javeriana. Bogotá, D.C., Colombia.

^3^ Laboratorio Asociaciones Suelo, Planta Microorganismo (LAMIC). Grupo de Investigación en Agricultura Biológica. Departamento de Biología. Facultad de Ciencias. Pontificia Universidad Javeriana. Bogotá, D.C., Colombia.

^4^ Facultad de Estudios Ambientales y Rurales. Departamento de Ecología y Territorio. Pontificia Universidad Javeriana. Bogotá, D.C., Colombia.

^5^ Facultad de Ciencias de la Salud. Universidad Anáhuac Campus Norte. México, D.F., México.

^6^ Departamento de Ciências Agrárias e Naturais. Universidade do Estado de Minas Gerais. Ituiutaba, Minas Gerais. Brazil.

^7^ Instituto Politécnico Nacional, Escuela Nacional de Ciencias Biológicas, Prolongación de Carpio y Plan de Ayala S/N, Col. Santo Tomás, México CDMX, C.P. 11340.

***Corresponding author**

**Aura M. Pedroza-Rodríguez,** Ph.D.

Professor

Pontificia Universidad Javeriana, Bogotá, D.C., Colombia

Facultad de Ciencias

Departamento de Microbiología. Unidad de Investigaciones Agropecuarias (UNIDIA) y Grupo de Biotecnología Ambiental e Industrial (GBAI)

Laboratorio de Microbiología Ambiental y de Suelos

Carrera 7^ma^ No 43-82, Edifício 50 Lab. 106

Código Postal: Bogotá 110-23

E-mail: [apedroza@javeriana.edu.co](mailto:apedroza@javeriana.edu.co)

Supplementary Material S1

| **Parameter** | **Unit** | **Treatment **** | | | | | | | | |
| --- | --- | --- | --- | --- | --- | --- | --- | --- | --- | --- |
|  |  | BC_500_ | BC_500_/PSB | | T1 | T2 | T3 | T4 | T5 | T6 |
| pH |  | 6.2±0.3 | | 6.0±0.6 | 7.44 | 7.59 | 7.41 | 7.65 | 7.65 | 7.73 |
| CE | dS m^-1^ | 0.58 | | 0.18 | 1.02 | 0.93 | 0.96 | 0.95 | 0.94 | 1.15 |
| Bulk density | g cc^-1^ | ND | | ND | 0.935 | 0.895 | 0.866 | 0.934 | 0.9 | 0.909 |
| CEC | Meq 100 g^-1^ | ND | | ND | 29 | 31.4 | 30.6 | 34.5 | 31.8 | 31.1 |
| Organic carbon | (%) | ND | | ND | 4.79 | 5.79 | 6.22 | 5.05 | 5.54 | 5.2 |
| Organic matter |  | ND | | ND | 8.26 | 9.98 | 10.7 | 8.71 | 9.55 | 8.96 |
| Total nitrogen |  | ND | | ND | 0.399 | 0.482 | 0.518 | 0.421 | 0.462 | 0.433 |
| C/N ratio |  | ND | | ND | 12 | 12 | 12 | 12 | 12 | 12 |
| Exchangeable potassium | mg kg^-1^ | 11.9* | | 6.8* | 490 | 500 | 520 | 510 | 490 | 510 |
| Interchangeable sodium |  | 46.0* | | 18.0* | 280 | 270 | 280 | 260 | 280 | 260 |
| Calcium |  | 54.0* | | 11.3* | 6600 | 7050 | 6880 | 7080 | 6920 | 6720 |
| Magnesium |  | 6.8* | | 2.75* | 144 | 135 | 151 | 137 | 139 | 123 |
| Iron |  | 0.085* | | 0.065* | 6.12 | 2.73 | 3.36 | 3.7 | 1.89 | 8.41 |
| Boro Boron |  | 0.4* | | 0.053* | 1.73 | 1.73 | 1.76 | 1.73 | 1.68 | 1.96 |
| Total phosphorus *** |  | ND | | ND | 1773.6 | 1839 | 1923.8 | 2194.3 | 1786.9 | 1589.6 |
| Extractable phosphorus |  | ND | | ND | 159 | 161 | 148 | 171 | 179 | 168 |
| Soluble phosphorus |  | 46.3* | | 21.9* | 13 | 13.2 | 12.5 | 14.7 | 12.2 | 16.6 |
| Ammonium |  | 1.26* | | 0.480* | 69.5 | 81.6 | 83 | 76.1 | 77.4 | 80.1 |
| Nitrates |  | 0.75* | | 0.7* | 31.7 | 35.8 | 33.2 | 32.8 | 35 | 28.9 |
| Total PSB count | Log_10_ CFU g^-1^ | ND | | 5.6 | 4.7 | 4.4 | <100 | <100 | <100 | <100 |
| *Pseudomonas* sp. count |  | ND | | 5.4 | 4.5 | 4.0 | <100 | <100 | <100 | <100 |
| *Serratia* sp. count |  | ND | | 5.3 | 4.1 | 3.7 | <100 | <100 | <100 | <100 |
| *Kosakonia* sp. count |  | ND | | 5.1 | 3.6 | 4.1 | <100 | <100 | <100 | <100 |

BC: biochar: BC/PSB: biochar and phosphate solubilizing bacteria; T1: Soil + Abundagro® 70 Kg ha^-1^ + biofertilizer (BC_500_/PSB) at 5.0 % (w/w); T2 Soil + Abundagro® 70 Kg ha^-1^ + biofertilizer (BC_500_/PSB) at 2.0 % (w/w); T3 Soil + Abundagro® 70 Kg ha^-1^ + biochar (BC_500_) at 5.0 % (w/w); T4 Soil + Abundagro® 70 Kg ha^-1^ + biochar (BC_500_) at 2.0 % (w/w); T5 Soil + Abundagro® 70 Kg ha^-1^; T6 100 % Soil.

Table S1. Chemical, nutritional and microbiological analysis of soil fertilised and enriched with biochar and bacteria before sowing *A. cepa*.

Supplementaty Material S2

| **Parameter** | **Value** | **Unit** | **Analytical method** | **Reference** |
| --- | --- | --- | --- | --- |
| **Major components** | | | | |
| Total nitrogen | 0.24 | % | Summation |  |
| Lignin | 40.9 |  | Neutral detergent fiber method (NDF) | (Van Soest et al. 1991) |
| Cellulose | 39 |  |  |  |
| Hemicellulose | 15 |  |  |  |
| Extractable compounds | 5.1 |  | Difference |  |
| **Elements** | | | | |
| Potassium (K^+^) | 0.04 | % | Atomic absorption (NTC 5167) | (ICONTEC 2011a; ICONTEC 2011b) |
| Calcium (Ca^2+^) | 0.14 |  |  |  |
| Magnesium (Mg^2+^) | 10 | mg Kg^-1^ |  |  |
| Sodium (Na^+^) | 0.029 | % | Flame emission (NTC 5167) |  |
| Ammonium  (N-NH_4_^+^) | ND |  |  |  |
| **Sum of cations** | | | | |
| Total phosphorus (P_2_O_5_) | 0.06 | % | Colorimetric (NTC 5167) | (ICONTEC 2011a; ICONTEC 2011b) |
| Sulphates (S-SO_4_^=^) | 0.01 |  |  |  |
| Nitrates (N-NO_3_^-^) | ND |  |  |  |
| **Sum of anions** | | | | |
| Boron (B) | 34 | mg Kg^-1^ | Atomic absorption (NTC 5167) | (ICONTEC 2011a; ICONTEC 2011b) |
| Total Iron (pH Real) | 0.002 | % |  |  |
| Total manganese (Mn) | 10 | mg Kg^-1^ |  |  |
| Copper (Cu) | 5.5 |  |  |  |
| Zinc (Zn) | 122 |  |  |  |
| Aluminium (Al) | 2.3 |  |  |  |

Tabla S2. Complementary physical-chemical analysis for Caribbean pine sawdust (CPS)

Supplementaty Material S3

| **Description of treatment** | **pH** | **CE** | **Density (g cc^-1^) ^ns^** | **CO (%) ^ns^** | **MO (%) ^ns^** |
| --- | --- | --- | --- | --- | --- |
| Abundagro^®^ + 5 % Biochar + BPS | 7.69 ± 0.04 | 0.69 ± 0.06 | 0.87 ± 0.02 | 6.96 ± 0.33 | 12.0 ± 0.57 |
| Abundagro^®^ + 2 % Biochar + BPS | 7.23 ± 1.25 | 0.77 ± 0.16 | 0.87 ± 0.08 | 6.60 ± 1.03 | 11.4 ± 1.77 |
| Abundagro^®^ + 5 % Biochar alone | 7.70 ± 0.05 | 0.65 ± 0.07 | 0.84 ± 0.09 | 7.64 ± 2.24 | 13.1 ± 3.95 |
| Abundagro^®^ + 2 % Biochar alone | 7.69 ± 0.04 | 0.74 ± 0.113 | 0.86 ± 0.05 | 6.86 ± 0.95 | 11.8 ± 1.66 |
| Abundagro^®^ | 7.76 ± 0.035 | 0.67 ± 0.08 | 0.87 ±0.03 | 6.64 ± 0.55 | 11.5 ± 0.92 |
| Water | 7.58 ± 0.41 | 0.82 ± 0.08 | 0.88 ± 0.04 | 6.58 ± 0.46 | 11.2 ± 0.56 |
| CV (%) | 2.57 | 8.90 | 1.69 | 5.87 | 5.64 |
| **Description of treatment** | **N (%) ^ns^** | **K (mg Kg^-1^) ^ns^** | **Na (mg Kg^-1^)** | **Ca (mg Kg^-1^)** | **Mg (mg Kg^-1^)** |
| Abundagro^®^ + 5 % Biochar + BPS | 0.58 ± 0.03 | 538.3 ± 33.7 | 182.7 ± 13.8 | 5286.7 ± 228.6 | 127.2 ± 4.3 |
| Abundagro^®^ + 2 % Biochar + BPS | 0.55 ± 0.09 | 510.0 ± 49.8 | 190.7 ± 16.2 | 5240.0 ± 216.8 | 129.5 ± 9.1 |
| Abundagro^®^ + 5 % Biochar alone | 0.63 ± 0.189 | 548.3 ± 51.1 | 175.0 ± 7.9 | 5331.7 ± 215.5 | 120.8 ± 3.1 |
| Abundagro^®^ + 2 % Biochar alone | 0.57 ± 0.08 | 495.0 ± 53.2 | 181.3 ± 25.4 | 5216.7 ± 281.8 | 123.5 ± 3.7 |
| Abundagro^®^ | 0.55 ± 0.05 | 523.3 ± 48.0 | 162.0 ± 10.9 | 5438.3 ±253.3 | 120.7 ± 3.3 |
| Water | 0.55 ± 0.04 | 498.3 ± 59.1 | 176.3 ± 14.4 | 5345.0 ± 241.6 | 117.5 ± 5.8 |
| CV (%) | 5.59 | 4.17 | 5.40 | 1.51 | 3.63 |
| **Description of treatment** | **Fe (mg Kg^-1^)** | **B (mg Kg^-1^)** | **P tot (mg Kg^-1^)** | **P ext (mg Kg^-1^)** | **P sol (mg Kg^-1^)** |
| Abundagro^®^ + 5 % Biochar + BPS | 0.200 ± 0.15 | 1.63 ± 0.06 | 1464.5 ± 127.3 | 101.2 ± 12.1 | 6.43 ± 0.45 |
| Abundagro^®^ + 2 % Biochar + BPS | 0.177 ± 0.11 | 1.65 ± 0.07 | 1523.1 ± 125.8 | 93.4 ± 8.0 | 6.59 ± 0.91 |
| Abundagro^®^ + 5 % Biochar alone | 0.123 ± 0.023 | 1.62 ± 0.06 | 1469 ± 179.4 | 88.0 ± 5.8 | 7.31 ± 1.10 |
| Abundagro^®^ + 2 % Biochar alone | 0.1 ± 0.017 | 1.61 ± 0.06 | 1445.9 ± 167.3 | 88.6 ±7.0 | 6.20 ± 0.60 |
| Abundagro^®^ | 0.2 ± 0.165 | 1.63 ± 0.06 | 1333.2 ± 157.5 | 101.1 ± 10.7 | 7.55 ± 0.76 |
| Water | 0.140 ± 0.017 | 1.72 ± 0.03 | 1387.7 ± 255.2 | 93.1 ± 10.6 | 7.00 ± 0.72 |
| VC (%) | 24.68 | 2.51 | 4.67 | 6.16 | 7.70 |
| **Description of treatment** | **Mn (mg Kg^-1^)** | **Cu (mg Kg^-1^)** | **Zn (mg Kg^-1^)** | **S (mg Kg^-1^)** | **CEC** |
| Abundagro^®^ + 5 % Biochar + BPS | 61.2 ± 3.9 | 0.19 ± 0.06 | 9.46 ± 1.27 | 65.3 ± 12.2 | 29.6 ± 1.14 |
| Abundagro^®^ + 2 % Biochar + BPS | 64.7 ± 5.2 | 0.20 ± 0.09 | 10.31 ± 0.60 | 85.1 ± 15.1 | 29.4 ± 1.03 |
| Abundagro^®^ + 5 % Biochar alone | 58.2 ± 3.3 | 0.17 ± 0.05 | 9.90 ± 0.71 | 57.0 ± 8.3 | 29.8 ± 1.02 |
| Abundagro^®^ + 2 % Biochar alone | 55.3 ± 4.2 | 0.14 ± 0.05 | 9.22 ± 0.47 | 69.4 ± 17.1 | 29.1 ± 1.46 |
| Abundagro^®^ | 58.7 ± 4.6 | 0.25 ± 0.06 | 9.53 ± 1.32 | 58.3 ± 14.9 | 30.2 ± 1.39 |
| Water | 63.0 ± 10.5 | 0.15 ± 0.09 | 9.8 ± 0.71 | 79.3 ± 19.6 | 29.7 ± 1.08 |
| CV (%) | 5.75 | 22.58 | 3.93 | 16.34 | 1.26 |
| **Description of treatment** | **Sat Hum** | **Ca/Mg** | **Ca/K ^ns^** | **Mg/K** | **Ca/Mg/K ^ns^** |
| Abundagro^®^ + 5 % Biochar + BPS | 30.0 ± 1.3 | 25.3 ± 1.6 | 19.2 ± 1.4 | 0.79 ± 0.11 | 20.0 ± 1.4 |
| Abundagro^®^ + 2 % Biochar + BPS | 29.7 ± 1.4 | 24.7 ± 1.6 | 20.3 ± 2.7 | 0.82 ± 0.09 | 21.1± 2.7 |
| Abundagro^®^ + 5 % Biochar alone | 29.3 ± 1.2 | 26.8 ± 1.6 | 19.1 ± 2.1 | 0.67 ± 0.11 | 19.9 ± 2.2 |
| Abundagro^®^ + 2 % Biochar alone | 29.8 ± 1.1 | 25.6 ± 1.3 | 20.7± 2.4 | 0.81 ± 0.10 | 21.5 ± 2.5 |
| Abundagro^®^ | 29.2 ± 0.3 | 23.7 ± 0.9 | 20.4 ± 1.6 | 0.75 ± 0.06 | 21.1 ± 1.6 |
| Water | 29.3 ± 0.9 | 27.7 ± 1.9 | 21.2 ± 3.1 | 0.77 ± 0.09 | 22.0 ± 3.3 |
| CV (%) | 1.07 | 4.62 | 4.01 | 7.36 | 4.02 |
| **Description of treatment** | **Sat Mg** | **Sat Na** | **Sat K ^ns^** | **Sat Ca ^ns^** |  |
| Abundagro^®^ + 5 % Biochar + BPS | 3.54 ± 0.22 | 2.69 ± 0.19 | 4.66 ± 0.32 | 89.2 ± 0.40 |  |
| Abundagro^®^ + 2 % Biochar + BPS | 3.63 ± 0.22 | 2.83 ± 0.28 | 4.45 ± 0.55 | 89.1 ± 0.85 |  |
| Abundagro^®^ + 5 % Biochar alone | 3.34 ± 0.17 | 2.56 ± 0.14 | 4.73 ± 0.53 | 89.4 ± 0.69 |  |
| Abundagro^®^ + 2 % Biochar alone | 3.50 ± 0.16 | 2.71 ± 0.34 | 4.36 ± 0.46 | 89.5 ± 0.28 |  |
| Abundagro^®^ | 3.29 ± 0.10 | 2.34 ± 0.13 | 4.43 ± 0.32 | 89.9 ± 0.29 |  |
| Water | 3.26 ± 0.20 | 2.59 ± 0.23 | 4.31 ± 0.61 | 89.8 ± 0.88 |  |
| CV (%) | 4.33 | 6.39 | 3.66 | 0.37 |  |

Table S3. Nutritional characteristics and soil pH after cultivation of *A. cepa* L. in pots (final concentrations).

**Referencias**

ICONTEC (2011a) Norma Técnica Colombiana 5167 (segunda actualización). Productos para la industria agrícola. Productos orgánicos usados como abonos o fertilizantes y enmiendas de suelo. ICONTEC, Bogotá, Colombia

ICONTEC (2011b) Norma Técnica Colombiana 5167, Productos Para la Industria Agrícola. Productos Orgánicos Usados como Abonos o Fertilizantes y Enmiendas de Suelo, Bogotá – Colombia.

Van Soest PJ, Robertson JB, Lewis BA (1991) Methods for dietary fiber, neutral detergent fiber, and nonstarch polysaccharides in relation to animal nutrition. J Dairy Sci 74 (10):3583-3597. http://dx.doi.org/10.3168/jds.S0022-0302(91)78551-2
